# In-Depth Characterization of Full-Length Archived Viral Genomes after Nine Years of Posttreatment HIV Control

**DOI:** 10.1128/spectrum.03267-22

**Published:** 2023-01-24

**Authors:** Pauline Trémeaux, Frédéric Lemoine, Adeline Mélard, Marine Gousset, Faroudy Boufassa, Sylvie Orr, Valérie Monceaux, Olivier Gascuel, Olivier Lambotte, Laurent Hocqueloux, Asier Saez-Cirion, Christine Rouzioux, Véronique Avettand-Fenoel

**Affiliations:** a Université Paris Cité, Faculté de Médecine, Paris, France; b INSERM U1016, CNRS UMR8104, Institut Cochin, Paris, France; c AP-HP, Laboratoire de Virologie, Hôpital Cochin, Paris, France; d Institut Pasteur, Université de Paris, Unité Bio-informatique Evolutive, Paris, France; e Institut Pasteur, Université de Paris, Hub Bioinformatique et Biostatistiques, Paris, France; f INSERM CESP U1018, Université Paris Sud, Le Kremlin Bicêtre, France; g Institut de Systématique, Evolution, Biodiversité (ISYEB), UMR 7205–CNRS, Muséum National d’Histoire Naturelle, SU, EPHE UA, Paris, France; h AP-HP, Hôpital Bicêtre, UMR1184, Université Paris Saclay, Inserm, CEA, Le Kremlin Bicêtre, France; i Service des Maladies Infectieuses et Tropicales, CHR d’Orléans—La Source, Orléans, France; j Institut Pasteur, HIV Inflammation et Persistance, Paris, France; k AP-HP, Laboratoire de Microbiologie Clinique, Hôpital Necker-Enfants Malades, Paris, France; CMG, Hôpital Bicêtre, Le Kremlin Bicêtre; CMG, Hôpital Bicêtre, Le Kremlin Bicêtre; Maladies Infectieuses, CHU—Hôpital Robert Debré, Reims; Maladies Infectieuses, CHU—Hôpital Robert Debré, Reims; Maladies Infectieuses, CHU—Hôpital Robert Debré, Reim; Maladies Infectieuses, CHU—Hôpital Robert Debré, Reims; Hémato-Oncologie, Centre Hospitalier Henri Duffaut, Avignon; Hémato-Oncologie, Centre Hospitalier Henri Duffaut, Avignon; Maladies Infectieuses, CHITS Hôpital Sainte Musse, Toulon; Maladies Infectieuses, CHITS Hôpital Sainte Musse, Toulon; Maladies Infectieuses, CHITS Hôpital Sainte Musse, Toulon; Maladies Infectieuses, Hôpital Tenon, Paris; Maladies Infectieuses, Hôpital Tenon, Paris; Maladies Infectieuses, Hôpital Tenon, Paris; Maladies Infectieuses, Hôpital Tenon, Paris; Centre de Diagnostic et Thérapeutique, Hôtel Dieu, Paris; Centre de Diagnostic et Thérapeutique, Hôtel Dieu, Paris; Service d’Immunologie Clinique, Hôpitaux Universitaires Paris Centre—Hôtel Dieu; Service d’Immunologie Clinique, Hôpitaux Universitaires Paris Centre—Hôtel Dieu; Service d’Immunologie Clinique, Hôpitaux Universitaires Paris Centre—Hôtel Dieu; Unité Fonctionnelle de Pathologie Infectieuse, Hôtel Dieu, Paris; Unité Fonctionnelle de Pathologie Infectieuse, Hôtel Dieu, Paris; Médecine Interne, Hôpital Cochin, Paris; Maladies Infectieuses, Saint-Louis, Paris; Maladies Infectieuses, Saint-Louis, Paris; Maladies Infectieuses, Saint-Louis, Paris; Maladies Infectieuses, Saint-Louis, Paris; Immunologie Clinique, Hôpital Edouard Herriot, Lyon; Immunologie Clinique, Hôpital Edouard Herriot, Lyon; Immunologie Clinique, Hôpital Edouard Herriot, Lyon; Maladies Infectieuses et Tropicales, Hôpital de la Croix Rousse, Lyon; Maladies Infectieuses et Tropicales, Hôpital de la Croix Rousse, Lyon; Maladies Infectieuses et Tropicales, Hôpital de la Croix Rousse, Lyon; Maladies Infectieuses et Tropicales, Hôpital de la Croix Rousse, Lyon; Maladies Infectieuses et Tropicales, Hôpital de la Croix Rousse, Lyon; Maladies Infectieuses et Tropicales, Hôpital de la Croix Rousse, Lyon; Maladies Infectieuses, Bichat, Paris; Maladies Infectieuses, Bichat, Paris; Maladies Infectieuses, Bichat, Paris; Maladies Infectieuses, Hôpital Bretonneau, Tours; Maladies Infectieuses, Hôpital Bretonneau, Tours; Maladies Infectieuses, Hôpital Bretonneau, Tours; Maladies Infectieuses, Hôpital Gabriel-Montpied, Clermont-Ferrand; Maladies Infectieuses, Hôpital Gabriel-Montpied, Clermont-Ferrand; Infectiologie, CHU François Mitterrand, Dijon; Infectiologie, CHU François Mitterrand, Dijon; Infectiologie, CHU François Mitterrand, Dijon; Maladies Infectieuses, Hôpital Gustave Dron, Tourcoing; Maladies Infectieuses, Hôpital Gustave Dron, Tourcoing; Maladies Infectieuses, Hôpital Gustave Dron, Tourcoing; Maladies Infectieuses et Tropicales, Hôpital Charles Nicolle, Rouen; Maladies Infectieuses et Tropicales, Hôpital Charles Nicolle, Rouen; Maladies Infectieuses, Hôpital de La Source, Orléans; Maladies Infectieuses, Hôpital de La Source, Orléans; Maladies Infectieuses, Hôpital de La Source, Orléans; Service des Maladies Infectieuses, CH de Belfort—Montbéliard, Belfort; Service des Maladies Infectieuses, CH de Belfort—Montbéliard, Belfort; Service de Médecine Interne, CHU Poitiers—La Milétrie, Poitiers; Service de Médecine Interne, CHU Poitiers—La Milétrie, Poitiers; Service des Maladies Infectieuse et Tropicales, CH de Perpignan, Perpignan; Service des Maladies Infectieuse et Tropicales, CH de Perpignan, Perpignan; Service des Maladies Infectieuses, CHU Reims—Hôpital Robert Debré, Reims; Service des Maladies Infectieuses, CHU Reims—Hôpital Robert Debré, Reims; Service des Maladies Infectieuses, CHU Reims-Hôpital Robert Debré, Reims; Service de Médecine Interne, CHI Ballanger, Aulnay Sous-Bois; Service d’Hématologie, CH Sud-Francilien—Hôpital Gilles de Corbeil, Corbeil-Evry; Service de Médecine Interne-Maladies Infectieuses, CH de Marne la Vallée, Jossigny; Service de Médecine Interne-Maladies Infectieuses, CH de Marne la Vallée, Jossigny; Service Hématologie-Immunologie, Centre Hospitalier Victor Dupouy, Argenteuil; Service Hématologie-Immunologie, Centre Hospitalier Victor Dupouy, Argenteuil; Service des Maladies Infectieuses et Tropicales, CHI Villeneuve Saint Georges, Villeneuve Saint Georges; Service des Maladies Infectieuses et Tropicales, CHI Villeneuve Saint Georges, Villeneuve Saint Georges; Service de Dermatologie, Hôpital Saint-Jacques, Besançon; Service de Dermatologie, Hôpital Saint-Jacques, Besançon; Service de Médecine Interne, CHU de Besançon, Besançon; Service d’Immunopathologie Clinique, Hôpital Saint Louis, Paris; Service d’Immunopathologie Clinique, Hôpital Saint Louis, Paris; Service de Maladies Infectieuses et Tropicales, Hôpital Saint Louis, Paris; Service de Maladies Infectieuses et Tropicales, Hôpital Saint Louis, Paris; Service de Maladies Infectieuses et Tropicales, Hôpital Saint Louis, Paris; Service de Maladie Infectieuse, Hôpital Bellevue, Saint Etienne; Service de Maladie Infectieuse, Hôpital Bellevue, Saint Etienne; Service des Maladies Infectieuses, CHU—Hôpital Pellegrin, Bordeaux; Service des Maladies Infectieuses, CHU—Hôpital Pellegrin, Bordeaux; Service des Maladies Infectieuses, CHU—Hôpital Pellegrin, Bordeaux; Service des Maladies Infectieuses, CHU—Hôpital Pellegrin, Bordeaux; Service Tropicales, CHU—Hôpital Saint André, Bordeaux; Service de Médecine Interne et Maladies Tropicales, CHU—Hôpital Saint André, Bordeaux; Service de Médecine Interne et Maladies Tropicales, CHU—Hôpital Saint André, Bordeaux; Service de Médecine Interne et Maladies Tropicales, CHU—Hôpital Saint André, Bordeaux; Service de Médecine Interne et Maladies Tropicales, CHU—Hôpital Saint André, Bordeaux; Service des Maladies Infectieuses, Centre Hospitalier Annecy, Annecy; Service des Maladies Infectieuses, Centre Hospitalier Annecy, Annecy; Service de Médecine Interne, AP-HP—CHU de Bicêtre, Le Kremlin Bicêtre; Service de Médecine Interne, AP-HP—CHU de Bicêtre, Le Kremlin Bicêtre; Service de Médecine Interne, AP-HP—CHU de Bicêtre, Le Kremlin Bicêtre; Service de Médecine Interne, AP-HP—CHU de Bicêtre, Le Kremlin Bicêtre; Service des Maladies Infectieuses, AP-HP—CHU de Bicêtre, Le Kremlin Bicêtre; Service des Maladies Infectieuses, AP-HP—CHU de Bicêtre, Le Kremlin Bicêtre; Consultation d’Hématologie, AP-HP—CHU de Bicêtre, Le Kremlin Bicêtre; Service de Médecine Interne A, Hôpital Lariboisière, Paris; Service de Médecine Interne A, Hôpital Lariboisière, Paris; Service Hématologie, Hôpital Henri Duffaut, Avignon; Service de Maladies Infectieuses, CHI de Poissy-Saint Germain en Laye, Saint Germain en Lay; Service d’Infectiologie, CHITS Hopital Sainte Musse, Toulon; Service d’Infectiologie, CHITS Hopital Sainte Musse, Toulon; Service des Maladies Infectieuses, Hôpital d’Instruction des Armées Bégin, Saint Mandé; Service des Maladies Infectieuses, Hôpital d’Instruction des Armées Bégin, Saint Mandé; Département de Médecine Aigue Spécialisée, Hôpital Raymond Poincaré, Garches; Département de Médecine Aigue Spécialisée, Hôpital Raymond Poincaré, Garches; Unité de Maladies Infectieuses, Hôpital Jean Verdier, Bondy; Unité de Maladies Infectieuses, Hôpital Jean Verdier, Bondy; Consultation de Maladies Infectieuses, Centre Médical de l’Institut Pasteur, Paris; Consultation de Maladies Infectieuses, Centre Médical de l’Institut Pasteur, Paris; Service des Maladies Infectieuses, Hôpital Tenon, Paris; Service des Maladies Infectieuses, Hôpital Tenon, Paris; Service de Médecine Interne et Endocrinologie, Hôpital Avicenne, Bobigny; Service de Médecine Interne et Endocrinologie, Hôpital Avicenne, Bobigny; Service de Médecine Interne, Hôpital Antoine Béclère, Clamart; Service de Médecine Interne, Hôpital Antoine Béclère, Clamart; Consultation d’Immunologie Clinique et Infectiologie, Hôpital Hôtel Dieu, Paris; Consultation d’Immunologie Clinique et Infectiologie, Hôpital Hôtel Dieu, Paris; Consultation d’Immunologie Clinique et Infectiologie, Hôpital Hôtel Dieu, Paris; Consultation d’Immunologie Clinique et Infectiologie, Hôpital Hôtel Dieu, Paris; Consultation d’Immunologie Clinique et Infectiologie, Hôpital Hôtel Dieu, Paris; Consultation d’Immunologie Clinique et Infectiologie, Hôpital Hôtel Dieu, Paris; Service de Médecine Interne, Hôpital Foch, Suresnes; Service de Médecine Interne, Hôpital Foch, Suresnes; Service de Médecine Interne, Hôpital Foch, Suresnes; Service de Médecine Interne, Hôpital Louis Mourier, Colombes; Service de Médecine Interne, Hôpital Louis Mourier, Colombes; Service d’Immunologie Clinique, Hôpital Henri Mondor, Créteil; Service des Maladies Infectieuses, Hôpital Pitié-Salpêtrière, Paris; Service des Maladies Infectieuses, Hôpital Pitié-Salpêtrière, Paris; Service des Maladies Infectieuses, Hôpital Pitié-Salpêtrière, Paris; Service des Maladies Infectieuses, Hôpital Pitié-Salpêtrière, Paris; Service des Maladies Infectieuses, Hôpital Saint Antoine, Paris; Service des Maladies Infectieuses, Hôpital Saint Antoine, Paris; Service de Maladies Infectieuses et Tropicales, Hôpital Pierre Zobda-Quitman, Fort de France, Martinique; Service de Maladies Infectieuses et Tropicales, Hôpital Pierre Zobda-Quitman, Fort de France, Martinique; Service des Maladies Infectieuses et Tropicales, CHU Angers, Angers; Service des Maladies Infectieuses et Tropicales, CHU Angers, Angers; Service des Maladies Infectieuses et Tropicales, CHU Angers, Angers; Service de Pneumologie, CHU de Brest, Brest; Service de Pneumologie, CHU de Brest, Brest; Service de Pneumologie, CHU de Brest, Brest; Service des Maladies Infectieuses, CHU de Limoges, Limoges; Service des Maladies Infectieuses, CHU de Limoges, Limoges; Service d’Immunologie Clinique, HCL—Hôpital Edouard Herriot, Lyon; Service d’Immunologie Clinique, HCL—Hôpital Edouard Herriot, Lyon; Service de Maladies Infectieuses et Tropicales, HCL—Hôpital Edouard Herriot, Lyon; Service de Maladies Infectieuses et Tropicales, HCL—Hôpital Edouard Herriot, Lyon; Service Hématologie—CISIH, Hôpital Sainte Marguerite, Marseille; Service Hématologie—CISIH, Hôpital Sainte Marguerite, Marseille; Service Hématologie—CISIH, Hôpital Sainte Marguerite, Marseille; Consultation de Médecine Interne, Hôpital Européen Marseille, Marseille; Consultation de Médecine Interne, Hôpital Européen Marseille, Marseille; Service des Maladies Infectieuses, Hôpital Bichat Claude Bernard, Paris; Service des Maladies Infectieuses, Hôpital Bichat Claude Bernard, Paris; Service des Maladies Infectieuses, Hôpital Bichat Claude Bernard, Paris; Service de Médecine Interne, Hôpital de l’Hôtel Dieu, Nantes; Service de Médecine Interne, Hôpital de l’Hôtel Dieu, Nantes; Service de Médecine Interne Post-Urgence, Centre Hospitalier Départemental, La Roche sur Yon; Service des Maladies Infectieuses, CHU—Hôpital l’Archet, Nice; Service des Maladies Infectieuses, CHU—Hôpital l’Archet, Nice; Service Médecine Interne, CHU—Hôpital l’Archet, Nice; Service des Maladies Infectieuses, CHU—Hôpital Pontchaillou, Rennes; Service des Maladies Infectieuses, CHU—Hôpital Pontchaillou, Rennes; Service des Maladies Infectieuses, CHU—Hôpital Pontchaillou, Rennes; HUS—Hôpital Civil, Strasbourg; HUS—Hôpital Civil, Strasbourg; Service des Maladies Infectieuses, CHRU—Hôpital Bretonneau, Tours; Service des Maladies Infectieuses, CHRU—Hôpital Bretonneau, Tours; Service des Maladies Infectieuses, CHU—Hôpital Purpan, Toulouse; Service des Maladies Infectieuses, CHU—Hôpital Purpan, Toulouse; Service Médecine Interne, CHG de Montauban, Montauban; Service des Maladies Infectieuses, CHU—Hôpital de la Côte de Nacre, Caen; Service des Maladies Infectieuses, CHU—Hôpital de la Côte de Nacre, Caen; Service des Maladies Infectieuse, CHU Gabriel Montpied, Clermont Ferrand; Service de Maladies Infectieuses et Tropicales, CHU—Hôpital du Bocage, Dijon; Service de Maladies Infectieuses et Tropicales, CHU—Hôpital du Bocage, Dijon; Service Médecin Aigue Spécialisée, CHU—Hôpital Albert Michallon, Grenoble; Service Médecin Aigue Spécialisée, CHU—Hôpital Albert Michallon, Grenoble; Service des Maladies Infectieuses, CH—Hôpital Gustave Dron, Tourcoing; Service des Maladies Infectieuses, CH—Hôpital Gustave Dron, Tourcoing; Service de Maladies Infectieuses et Tropicales, CHU Nancy, Nancy; Service de Maladies Infectieuses et Tropicales, CHU Nancy, Nancy; Service de Maladies Infectieuses et Tropicales, CHU—Hôpital Charles Nicolle, Rouen; Service de Maladies Infectieuses et Tropicales, CHU—Hôpital Charles Nicolle, Rouen; Service de Maladies Infectieuses et Tropicales, CHU—Hôpital Charles Nicolle, Rouen; Service de Médecine Interne, CH René Arbeltier, Coulommiers; Service de Maladies Infectieuses-Médecine Interne, Hôpitaux Civils de Colmar, Colmar; Service de Maladies Infectieuses-Médecine Interne, Hôpitaux Civils de Colmar, Colmar; Service de Pneumo-Phtisiologie, Centre Hospitalier Sud Réunion—Hôpital de St Pierre, Saint Pierre, La Réunion; Service Immunologie Clinique, Centre Hospitalier Félix Guyon, Saint-Denis, Ile de la Réunion; Service de Médecine Interne, Hôpital Beaujon, Clichy; Service de Médecine Interne, CHR Orléans—Hôpital Porte Madeleine, Orléans; Service de Médecine Interne, CHR Orléans—Hôpital Porte Madeleine, Orléans; Service de Maladies Infectieuses et Tropicales, Hôpital Orléans la Source, Orléans; Service de Maladies Infectieuses et Tropicales, Hôpital Orléans la Source, Orléans; Service des Médecine Interne, Centre Hospitalier Intercommunal, Créteil; Service des Médecine Interne, Centre Hospitalier Intercommunal, Créteil; Service de Médecine A, CASH—Hôpital Max Fourestier, Nanterre; Service de Médecine Interne 2, Hôpital Ambroise Paré, Boulogne; Service d’Hématologie Clinique VIH, Centre Hospitalier de Mulhouse, Mulhouse; Service de Pneumologie, CHI Les Hôpitaux du Léman, Thonon les Bains; Service Médecine-Gastroentérologie, Centre Hospitalier René Dubos, Pontoise; Service Médecine-Gastroentérologie, Centre Hospitalier René Dubos, Pontoise; Service de Médecine Interne, Centre Hospitalier de Saint Nazaire, Saint Nazaire; Service de Médecine Interne, CHR Metz Thionville—Hôpital Notre Dame de Bon Secours, Metz; Service de Médecine Interne, CHR Metz Thionville—Hôpital Notre Dame de Bon Secours, Metz; Service de Dermatologie, Hôpital Beauregard, Thionville; Service de Médecine Interne, Hôpital Prosper Chubert, CHBA, Vannes; Service de Médecine Interne, Hôpital Simone Veil, Eaubonne; Service de Médecine Interne, Hôpital Simone Veil, Eaubonne; Service des Maladies Infectieuses et Tropicales, CHU Caremeau, Nîmes; Service des Maladies Infectieuses et Tropicales, CHU Caremeau, Nîmes; Service de Pneumologie, CH de Cornouaille—Hôpital Laennec, Quimper; Service de Pneumologie, CH de Cornouaille—Hôpital Laennec, Quimper; Service d’Hématologie, Maladie Infectieuses, CH Bretagne Sud, Lorient; Service d’Hématologie, Maladie Infectieuses, CH Bretagne Sud, Lorient; Service Médecine Interne, CH de Cornouaille—Hôpital Laennec, Quimper; Service Médecine Interne, CH de Cornouaille—Hôpital Laennec, Quimper; Service des Maladies Infectieuses et Tropicales, CH Saint Malo, Saint Malo; Service des Maladies Infectieuses et Tropicales, CH Saint Malo, Saint Malo; Service de Maladies Infectieuses et Tropicales, Centre Hospitalier Le Mans, Le Mans; Service de Maladies Infectieuses et Tropicales, Centre Hospitalier Le Mans, Le Mans; Kumamoto University

**Keywords:** posttreatment HIV controller, HIV reservoirs, provirus, defective proviruses, next-generation sequencing, posttreatment HIV controllers, ultradeep sequencing, proviruses

## Abstract

In the search for control of human immunodeficiency virus type 1 (HIV-1) infection without antiretroviral therapy, posttreatment controllers (PTCs) are models of HIV remission. To better understand their mechanisms of control, we characterized the HIV blood reservoirs of 8 PTCs (median of 9.4 years after treatment interruption) in comparison with those of 13 natural HIV infection controllers (HICs) (median of 18 years of infection) and with those of individuals receiving efficient antiretroviral therapy initiated during either primary HIV infection (PHIs; *n* = 8) or chronic HIV infection (CHIs; *n* = 6). This characterization was performed with single-genome amplification and deep sequencing. The proviral diversity, which reflects the history of past viral replication, was lower in the PTCs, PHIs, and aviremic HICs than in the blipper HICs and CHIs. The proportions of intact and defective proviruses among the proviral pool in PTCs were not significantly different from those of other groups. When looking at the quantities of proviruses per million peripheral blood mononuclear cells (PBMCs), they had similar amounts of intact proviruses as other groups but smaller amounts of defective proviruses than CHIs, suggesting a role of these forms in HIV pathogenesis. Two HICs but none of the PTCs harbored only proviruses with deletion in *nef*; these attenuated strains could contribute to viral control in these participants. We show, for the first time, the presence of intact proviruses and low viral diversity in PTCs long after treatment interruption, as well as the absence of evolution of the proviral quasispecies in subsequent samples. This reflects low residual replication over time. Further data are necessary to confirm these results.

**IMPORTANCE** Most people living with HIV need antiretroviral therapy to control their infection and experience viral relapse in case of treatment interruption, because of viral reservoir (proviruses) persistence. Knowing that proviruses are very diverse and most of them are defective in treated individuals, we aimed to characterize the HIV blood reservoirs of posttreatment controllers (PTCs), rare models of drug-free remission, in comparison with spontaneous controllers and treated individuals. At a median time of 9 years after treatment interruption, which is unprecedented in the literature, we showed that the proportions and quantities of intact proviruses were similar between PTCs and other individuals. Unlike 2/7 spontaneous controllers who harbored only *nef*-deleted proviruses, which are attenuated strains, which could contribute to their control, no such case was observed in PTCs. Furthermore, PTCs displayed low viral genetic diversity and no evolution of their reservoirs, indicating very low residual replication, despite the presence of intact proviruses.

## INTRODUCTION

Human immunodeficiency virus type 1 (HIV-1) infection became a chronic disease in patients under long-term antiretroviral therapy (ART) to control viral replication. Because of the existence of viral reservoirs, most people living with HIV-1 (PWH) experience viral rebound following antiretroviral treatment interruption (TI), whether they have been treated since the primary infection (primary HIV infection patient [PHI]) or the chronic stage (chronic HIV infection patient [CHI]), with rates of 87% and 96%, respectively, at 6 months after TI (1). Nevertheless, in rare cases, viral replication remains controlled for several years after ceasing ART. Most of these individuals, known as posttreatment controllers (PTCs), have been treated since primary infection, such as in the French ANRS-VISCONTI cohort (2). They differ from the rare individuals, known as natural HIV infection controllers (HICs), who maintain low or undetectable viral loads without treatment (<1%) (3, 4). In this context, current research on HIV remission aims at finding therapeutic alternatives that do not require the lifelong intake of ART. As PTCs constitute models of HIV infection control without ART, a better understanding of the viral and host parameters responsible for this control may help with the development of therapeutic strategies benefiting all PWH.

In HICs, an overrepresentation of protective HLA alleles, such as HLA-B*27 and B*57, has been observed, as well as a high cytotoxic CD8 T cell response (3). This has not been observed in PTCs, who instead present high expression of HLA-B*35, an allele associated with a faster progression toward AIDS in the absence of ART (2, 3). Contrary to HICs, PTCs also often exhibit symptomatic primary infection (1, 2), and these differences suggest that different mechanisms might occur in PTCs (4). Viral characteristics might also contribute to the mechanism of control of HIV infection in these two groups. For example, both PTCs and HICs have a small viral reservoir, as reflected by their particularly low levels of blood HIV DNA (2, 5, 6) or cell-associated HIV RNA (7). However, although necessary, a small reservoir size does not appear to be sufficient to induce viral control, as most individuals treated since the primary infection display low levels of total HIV DNA (8) but less than 10% of them might become PTCs after TI (1, 2, 9). The quality of the total HIV DNA, determined by considering the proportions of intact versus defective genomes within the proviral pool in these persons, could be important. Indeed, previous studies have shown that during ART-controlled infection, most of the HIV DNA corresponds to defective genomes containing large deletions, frameshifts, or apolipoprotein B mRNA-editing catalytic polypeptide-like (APOBEC)-induced hypermutations (10–13). Regarding HICs and PTCs, data on their proviral landscape is very limited, but one hypothesis is that a lower proportion and/or level of intact genomes might contribute to the control of infection. Genetic diversity is another viral factor that might contribute to the control of infection: a low diversity might facilitate the control by the immune system, while a higher one might help in escaping it. Regarding HICs, Jiang et al. recently showed that they were characterized by low levels of intact HIV DNA in specific integration sites (14). In HICs, viral genetic diversity appears correlated to the level of viral replication (15, 16). Information concerning PTCs is limited to a study by Sharaf et al. that included PTCs with a generally shorter period of control than those of the ANRS-VISCONTI cohort. These PTCs displayed lower intact proviral loads than noncontrollers (NCs), although with similar overall proportions (17). Their proviral landscape had not evolved at a median of 1.4 years after TI. It is currently unclear whether PTCs maintain a low diversity several years after treatment interruption, similar to PHIs (18), or whether this diversity increases after several years, similar to most untreated individuals.

In the present study, we aimed to investigate the proviral genome landscape in a group of PTCs with durable control of infection. To this end, we determined (i) the viral genetic diversity and (ii) the presence and proportions of intact and defective archived HIV genomes in blood samples from PTCs several years after TI, in comparison with those of HICs and of two groups of patients receiving efficient ART (since primary infection [PHIs] and since chronic infection [CHIs]).

## RESULTS

### Patients and samples.

To study the contribution of viral factors to HIV remission, we studied the archived HIV genomes in blood samples from four groups of PWH, including 8 early-treated PTCs from the ANRS-VISCONTI cohort (2), 13 HICs from the French multicenter ANRS-CODEX cohort (19), 8 PHIs, and 6 CHIs (Table 1). All PTCs had been treated during primary infection (<3 months after infection) and for a median time of 4.0 years (range, 1.1 to 16.8 years) (Table 1). The median time between the TI and the studied-sample date was 9.4 years (range, 1.2 to 13.8 years), and the median blood HIV DNA load was 1.96 log copies/10^6^ peripheral blood mononuclear cells (PBMCs) (range, 0.52 to 2.60 log copies/10^6^ PBMCs). Three PTCs had a second blood sample analyzed, with a median time of 5.2 years between the sampling dates. Except for the longitudinal part of the study, only the first sample of each PTC was included. HICs had been infected by HIV for a median time of 18 years at the time of sampling (range, 7 to 29 years), without ART. Their median blood HIV DNA load was 2.30 log copies/10^6^ PBMCs (Table 1). The PHI and CHI groups included PWH with undetectable viremia under ART initiated either during primary infection or the chronic stage, respectively. Treatment of the PHI group was initiated as early as that of the PTC group (Table 1), and these patients had been under efficient ART for a median time of 5.2 years at the time of sampling. Individuals in the CHI group had initiated ART at a median time of 3.8 years after the diagnosis (range, 2.5 to 13.3 years), and they had been treated for a median time of 9.2 years at the time of sampling.

**TABLE 1:** Patient characteristics^*a*^

Group	Individual	Former ID^*b*^	HLA allele	Age at diagnosis (yrs)	Yr of HIV diagnosis	Plasma HIV RNA peak (log copies/mL)^*c*^	Sample yr	Duration of ART (yrs)	Value on the sample date for:				Remarks
									Time since TI (yrs)	Plasma HIV RNA (copies/mL)^*d*^	Total HIV DNA (log copies/10^6^ PBMCs)	CD4 T-cell count/μL	
PTC	180001^*e*^	OR1	B*35/B*49	31	1996	4.32	2011	6.7	8.7	2	2.19	NA	
							2017		13.9	<8	1.88	1,019	
	180002^*e*^	OR2	B*35/B*45	31	2001	6.79	2011	2.2	7.5	2	1.82	528	
							2015		11.8	<10	0.52	759	
	180003^*e*^	OR3	B*14/B*49	31	1996	3.38 (after PEP)	2013	7.1	9.4	115	2.06	429	
							2018		15.2	1,278	1.96	375	ART resumption in 2018 after the second sample (180003b)
	098001	LY1	B*07/B*57	55	2001	4.87	2010	1.9	7.2	6	1.25	701	ART resumption in 2011 (cancer)
	070001		B*35/B*53	41	1996	3.26	2014	16.8	1.2	107	2.60	1,709	ART resumption in 2015
	041001		B*15/B*38	38	1998	5.68	2014	2.4	13.8	3	<0.78	682	
	200001	KPV	B*13/B*35	37	2001	3.03	2011	1.1	8.9	200	<2.15	658	ART resumption in 2015
	073001	Case A	B*15/B*41	0^*b*^	1996 or early 1997	6.34	2015	5.5	12.0	2	2.23	727	

	Median (IQR)			34 (31–38)		4.60 (3.35–5.85)		4.0 (1.9–6.7)	9.4 (7.4–12.5)	8 (3–111)	1.96 (1.82–2.19)	692 (561–751)	

HIC-a	CODa1		B*14/B*27	46	1993	4.00	2014	No ART		3	2.18	405	
	CODa2		B*13/B*70	23	1991	2.48	2014	No ART		3	2.46	844	
	CODa3		B*51/B*39	33	1996	<2.70	2014	No ART		<2	2.15	1,184	
	CODa4		B*14/B*27	33	1985	<3.00	2014	No ART		<2	2.26	834	ART initiation in 2017
	CODa5		B*05/B*70	40	2008	1.85	2015	No ART		71	1.98	757	
	CODa6		B*17/B*70	26	2008	<1.60	2018	No ART		<2	2.30	1,108	

	Median (IQR)			33 (28–38)		2.59 (2.01–2.93)				3 (1–3)	2.22 (2.16–2.29)	839 (776–1,042)	

HIC-b	CODb1		B*14/B*49	45	2004	2.03	2015	No ART		742	2.91	868	ART initiation in 2017
	CODb2		B*14/B*27	49	1988	2.98	2014	No ART		156	2.50	487	ART initiation in 2016
	CODb3		B*16/B*17	27	1995	2.90	2014	No ART		428	2.79	463	ART initiation in 2015
	CODb4		B*08/B*27	23	2002	2.63	2015	No ART		280	2.54	607	ART initiation later in 2015 (pregnancy)
	CODb5		B*52/B*52	21	1996	2.58	2014	No ART		245	2.29	546	
	CODb6		B*07/B*57	29	1998	2.74	2014	No ART		16	2.14	550	
	CODb7		B*12/B*42	54	2009	2.09	2017	No ART		87	2.74	450	ART initiation in 2019

	Median (IQR)			29 (25–47)		2.63 (2.34–2.82)				245 (122–354)	2.54 (2.40–2.77)	546 (475–579)	

All HICs	Median (IQR)			33 (26–45)		2.63 (2.09–2.90)				71 (3–245)	2.30 (2.18–2.54)	607 (487–844)	
PHI	PHI1		B*40/B*51	50	2005	4.93	2010	4.9	ART ongoing	10	1.86	941	
	PHI2		B*44/B*47	36	1999	3.82	2013	13.8	ART ongoing	10	1.95	985	
	PHI3		B*39/B*40	34	2007	5.57	2010	3.3	ART ongoing	3	1.72	748	
	PHI4		B*27/B*40	32	2003	4.89	2008	5.4	ART ongoing	4	2.1	690	
	PHI5		B*49/B*53	43	2007	6.18	2010	3.2	ART ongoing	3	2.02	1,156	
	PHI6		B*07/B*56	36	2005	4.61	2012	6.2	ART ongoing	2	2.02	836	
	PHI7		B*15/B*51	42	2008	6.38	2012	3.3	ART ongoing	2	2.20	742	
	PHI8		B*35/B*49	42	1998	>5.90	2012	13.5	ART ongoing	3	1.43	847	

	Median (IQR)			39 (36–42)		5.25 (4.82–5.97)		5.2 (3.3–8.0)		3 (3–5)	1.99 (1.83–2.04)	842 (747–952)	

CHI	CHI1		NA	40	2003	5.39	2014	8.7	ART ongoing	<20	2.76	824	
	CHI2		NA	28	2005	5.15	2015	6.2	ART ongoing	<20	3.09	1,410	
	CHI3		NA	21	1994	2.30	2015	17.0	ART ongoing	<20	2.88	1,087	
	CHI4		NA	40	2007	5.24	2014	4.5	ART ongoing	<20	3.50	1,075	
	CHI5		NA	27	1994	4.73	2014	13.6	ART ongoing	<20	2.72	1,036	
	CHI6		NA	18	1991	4.91	2013	9.6	ART ongoing	<20	2.84	652	

	Median (IQR)			28 (23–37)		5.03 (4.78–5.22)		9.2 (6.8–12.6)			2.86 (2.78–3.04)	1,056 (877–1,084)	

a ART, antiretroviral therapy; TI, treatment interruption; CHI, chronic stage; HIC, natural HIV controller; NA, not available; PEP, postexposure prophylaxis; PHI, primary infection stage; PTC, posttreatment controller; IQR, interquartile range. HIC-a (aviremic) individuals were defined as having more than 50% of their viral loads from the 5 years before the sample date below 50 copies/mL and none above 400 copies/mL, while HIC-b (blipper) individuals did not meet those criteria.

b Former identification number (ID) from the initial description of the ANRS-VISCONTI cohort (2), except for “case A,” a mother-to-child transmission, 6-week prophylaxis at birth, ART initiated at 3 months of age (22).

c Value before ART initiation or between the diagnosis and the inclusion in the cohort for the HIC group.

d Quantified using an ultrasensitive technique, except for the CHI group or in case of a positive result with the regular technique.

e A second sample was analyzed for 3 PTCs, identified as 180001b, 180002b, and 180003b on the further data.

### PTCs, PHIs, and aviremic HICs have lower viral diversity than CHIs.

**FIG 1:**
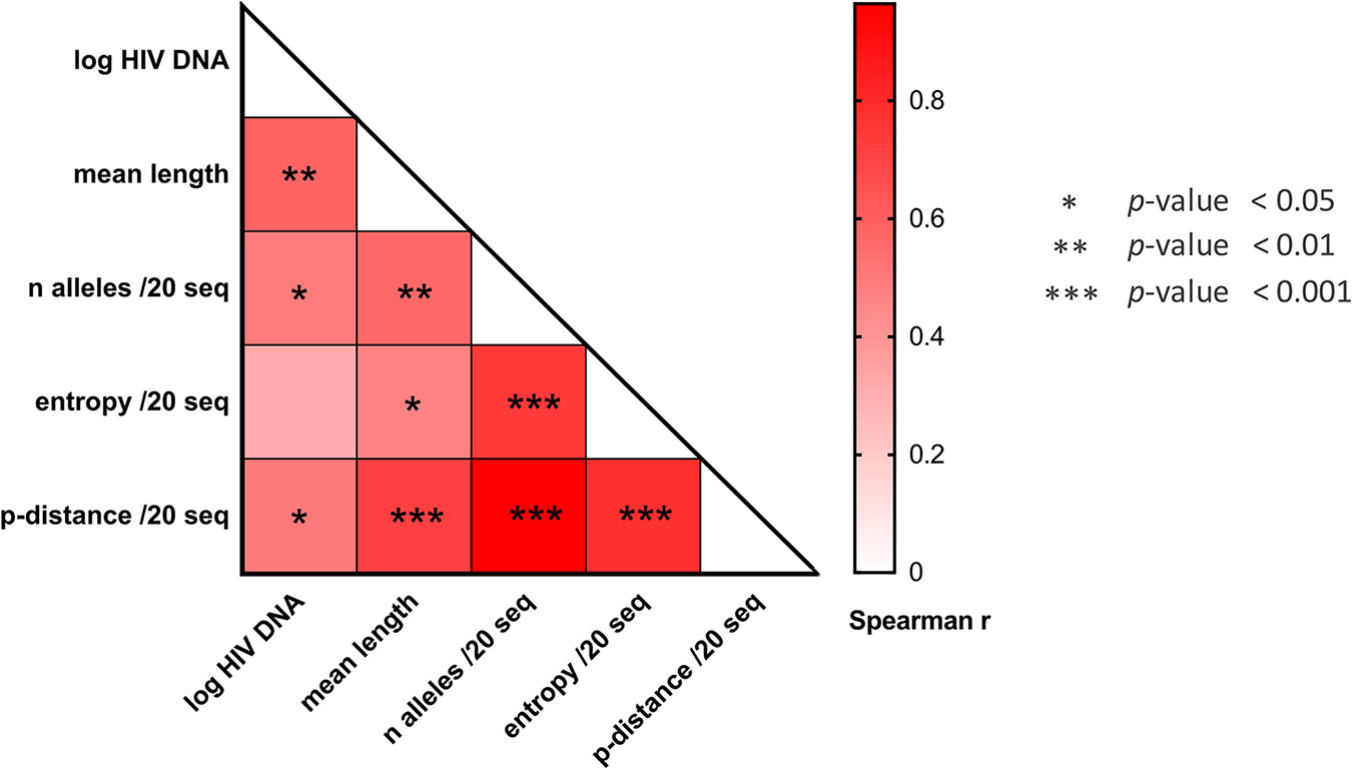
Correlation of the genetic diversity indexes. Viral diversity was calculated by several indexes and based on 24,091 nondefective *RT* sequences from 32 individuals: the mean branch length, the mean number of alleles per nucleotide site, the mean entropy, and the mean *p*-distance. A random sampling of 20 sequences without replacement was performed to calculate these indexes, except for the mean branch length; the final results are the mean values of 1,000 repetitions. Correlations between all diversity indexes and between each diversity index and the HIV DNA viral load (log copies/10^6^ PBMCs) were calculated using Spearman’s coefficients and considered significant when the *P* value was <0.05.

**FIG 2:**
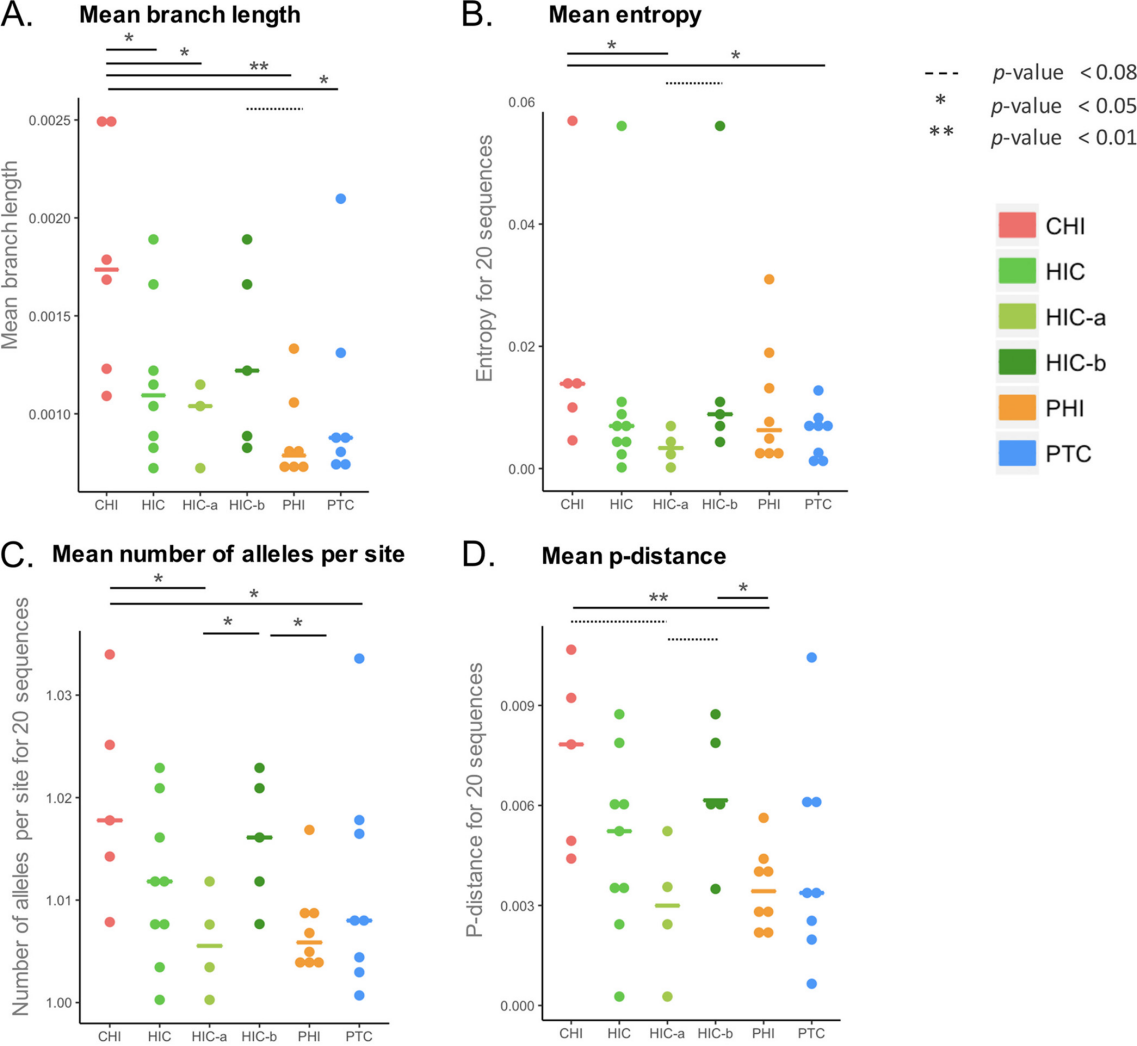
Viral diversity of HIV DNA based on the *RT* gene estimated by different indexes. A 700-bp amplicon on the *RT* gene was amplified in HIV DNA genomes and sequenced using a 454 GS Junior. Sequences were aligned with Clustal, and phylogenetic trees were constructed with PhyML. Viral diversity was calculated by several indexes and based on 24,091 nondefective *RT* sequences. The indexes used were the branch length of the phylogenetic tree (samples with fewer than 4 nondefective haplotypes were excluded) (A), the entropy (B), the number of alleles per nucleotide site (C), and the *p*-distance (D). A random sampling of 20 sequences without replacement was performed for indexes B to D; the final results are the mean values of 1,000 repetitions. The mean value for each group is depicted with a colored horizontal line. The diversity was compared among groups using Wilcoxon tests. Significant differences (*P* < 0.05) are depicted with continuous lines above the graph, while trends (*P* < 0.05 and *P* < 0.08) are depicted with dotted lines. HICs were divided into two subgroups: HIC-a (aviremic) individuals had more than 50% of their viral loads from the 5 years before the sample date below 50 copies/mL and none above 400 copies/mL, while HIC-b (blippers) individuals did not meet those criteria.

To study the genetic diversity of the archived HIV genomes together with the presence of stop codons/hypermutations in the blood samples from the different groups of PWH, we first performed the sequencing of the reverse transcriptase (*RT*) region of the *pol* gene. Although this region is not the most variable in the HIV genome, it is one of those most commonly targeted by APOBEC (20) and, thus, appeared to be an acceptable compromise to study both diversity and genetic defects induced by APOBEC. *RT* gene diversity analysis was performed on blood samples from 8 PTCs, 10 HICs, 8 PHIs, and 6 CHIs (Table S1 in the supplemental material). A total of 25,154 *RT* sequences were obtained for the 32 samples (median sequencing depth [interquartile range {IQR}], 180 [54 to 719]), including 24,091 nondefective sequences. Given that there is no “gold standard” method to express the genetic diversity of viral quasispecies (21), we used several indexes, including mean branch length, entropy, number of alleles per nucleotide site, and *p*-distance, to compare the proviral diversity. Overall, these indexes were correlated with each other (Fig. 1) and gave concordant results for comparison between groups (Fig. 2). The diversities were similar among the PTC and PHI groups, regardless of the index used, and significantly lower in median values than those of the CHI group, although heterogeneity was observed among each group of PWH. Of note, PTC 073001 had the highest genetic diversity among the PTC group, which can be explained by a history of past viral replication after mother-to-child transmission and before a sustained period of control over the last decade (22). The HIC group displayed no significant difference from PHIs and PTCs nor from CHIs, probably because of the high interindividual variability in this group. We thus defined subgroups among HICs: “aviremic” individuals (HIC-a) had more than 50% of their viral loads from the 5 years before the sample date below 50 copies/mL and none above 400 copies/mL, and “blippers” (HIC-b group) did not meet those criteria (19). The median blood HIV DNA loads were 2.22 and 2.54 log copies/10^6^ PBMCs for the HIC-a and the HIC-b groups, respectively (Table 1). The median times since infection were similar between subgroups. PHIs and PTCs showed proviral genetic diversities similar to that of the HIC-a group. In addition, the PHI and HIC-a groups displayed lower genetic diversities than the HIC-b group, but the difference between PTCs and the HIC-b group was not significant. There was no difference in genetic diversity between the HIC-b and CHI groups. Finally, all diversity indexes but entropy were significantly correlated with the HIV DNA load (*P < *0.05) (Fig. 1). Overall, our results indicate that there was no difference in genetic diversity among the PTC, PHI, and HIC-a groups, that each of these groups had a lower diversity than the CHI group, and that genetic diversity was correlated with the total HIV DNA load.

### PTCs have proportions of intact proviruses similar to those of other groups.

**FIG 3:**
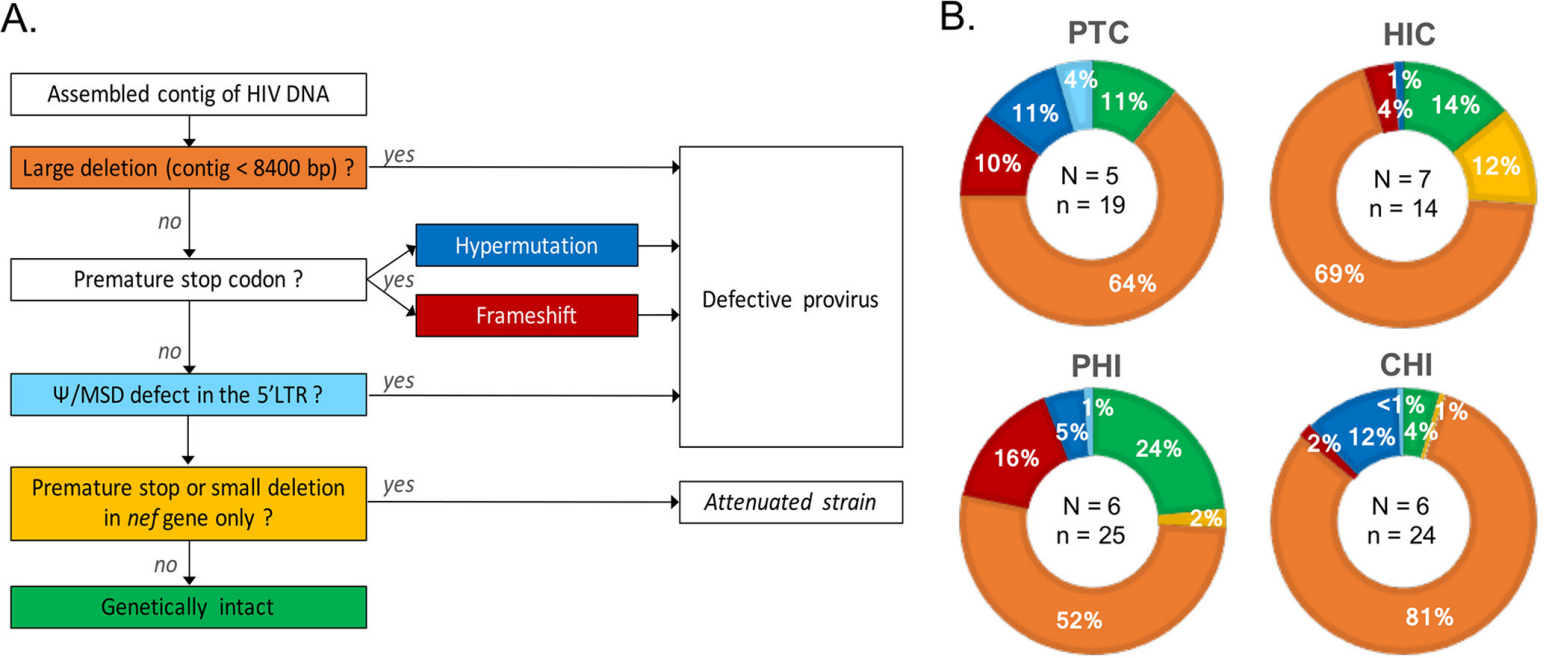
Identification of intact sequences and the different types of defective sequences and their proportions. HIV DNA genomes were amplified from the 5′LTR to the 3′LTR after limiting dilution and sequenced using a MiSeq instrument. Complete genomic sequences were assembled using Spades. (A) Process of identification of genetically defective and intact proviruses. A sequential approach was used. Premature stop codons due to hypermutations and frameshifts were detected using pairwise alignment implemented in Goalign. MSD defects were visually identified by alignment of genomic sequences and comparison to HIV compendium sequences. (B) Mean proportions of each type of HIV DNA genome in each group of patients: *N* is the number of patients in the group, and *n* is the mean number of full-length sequences per individual.

To evaluate the prevalence of stop codons and hypermutations, we first considered the sequences of the *RT* gene used for the HIV DNA diversity analysis. These defects were detected in 25%, 20%, 25%, and 33% of individuals from the PTC, HIC, PHI, and CHI groups, respectively (Table S1). In order to further explore the defectiveness of proviruses, we expanded the sequencing target length to nearly the full-length genome (from the 5′ long terminal repeat [LTR] to the 3′LTR, 8.9 kb) and characterized the other types of genetic defects (large deletion, premature stop codon, and Ψ/MSD defect in the 5′ LTR) (Fig. 3A). Besides intact and defective HIV genomes, sequences with only a defect in the *nef* gene were classified as “attenuated strains”, since the virus can replicate in the absence of the accessory Nef protein, although with decreased infectivity (23). Because the quantity of biological material was limited, the complete HIV DNA genome analysis could only be performed on samples from 5 PTCs, 7 HICs, 6 PHIs, and 6 CHIs (for two additional PTC and two additional PHI participants, fewer than seven full-genome sequences were obtained, and these were excluded from analyses) (Table S2). A total of 491 complete HIV DNA sequences were then obtained for the 24 samples (median number of sequences per sample [IQR], 21 [18 to 24]).

**FIG 4:**
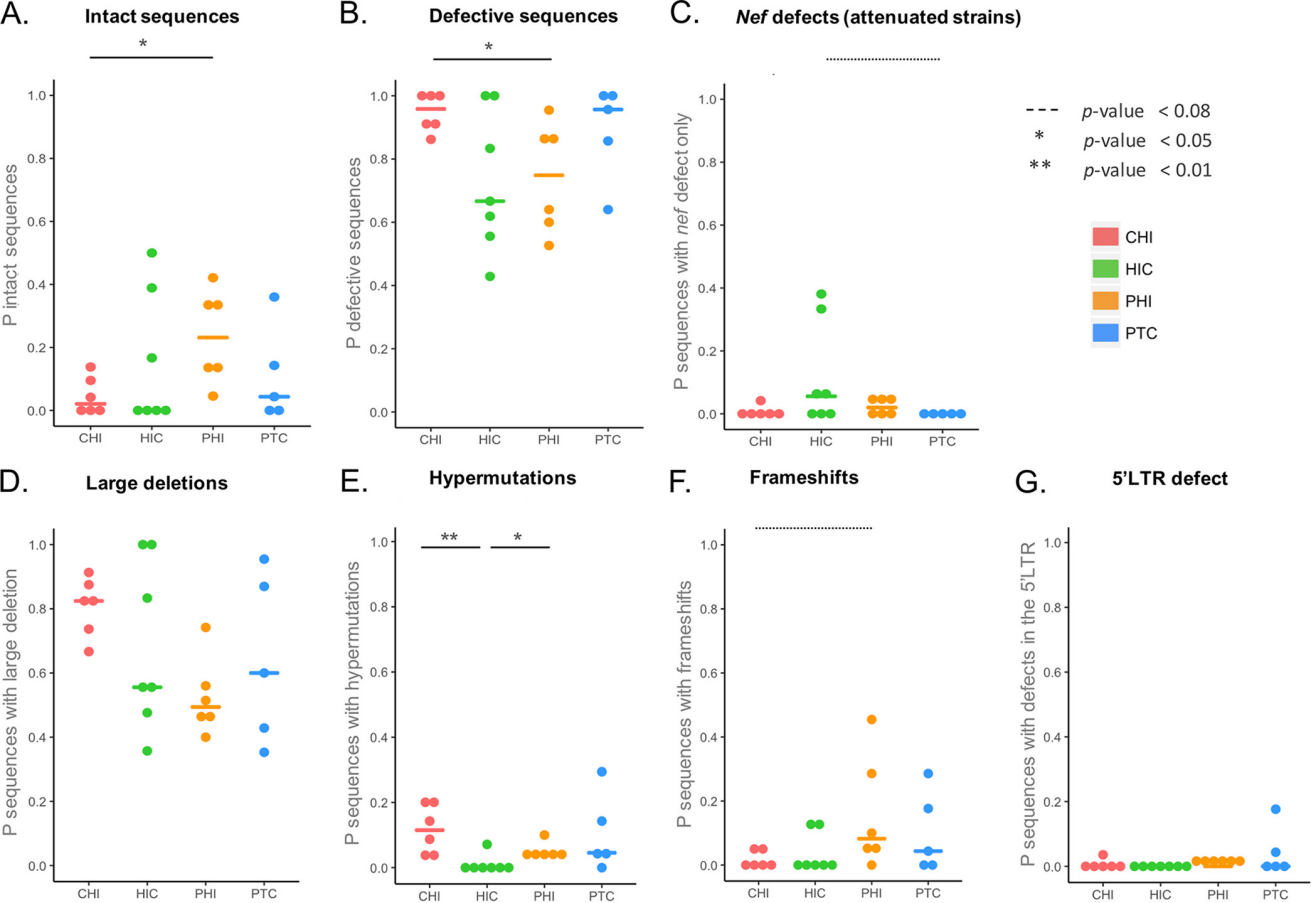
Proportions of each type of full-length HIV DNA genome. HIV DNA genomes were amplified from the 5′LTR to the 3′LTR and sequenced using a MiSeq instrument. Complete genomic sequences were assembled using Spades. Genetic defects were sequentially identified as follows: large deletions (D), APOBEC-induced hypermutations (E), frameshifts (F), and defects in the Ψ/MSD regions of the 5′LTR (G). Sequences with a defect in the *nef* gene only were considered attenuated strains (C), and the remaining sequences were inferred to be intact (A). The mean value for each group is depicted with a colored horizontal line. The proportions of each type of provirus were compared among groups using Wilcoxon tests. Significant differences (*P* < 0.05) are depicted with continuous lines above the graphs, while trends (*P* values between 0.05 and 0.08) are depicted with dotted lines. (A) The median proportions (P) of intact proviruses were 2% in CHIs, 0% in HICs, 22% in PHIs, and 4% in PTCs. (B) The median proportions of defective proviruses (sum of large deletions, hypermutations, frameshifts, and 5′LTR defects) were 96% in CHIs, 67% in HICs, 75% in PHIs, and 96% in PTCs. (C) The median proportions of proviruses with defects in the *nef* gene only (attenuated strains) were 0% in CHIs, 6% in HICs, 2% in PHIs, and 0% in PTCs. (D) The median proportions of proviruses with large deletions were 82% in CHIs, 56% in HICs, 49% in PHIs, and 60% in PTCs. (E) The median proportions of proviruses with hypermutations were 11% in CHIs, 0% in HICs, 4% in PHIs, and 5% in PTCs. (F) The median proportions of proviruses with frameshifts were 0% in CHIs, 0% in HICs, 8% in PHIs, and 4% in PTCs. (G) The median proportions of proviruses with 5′LTR defects were 0% in all four groups.

Overall, intact proviruses were detected in PBMCs for 3/5 PTCs, and their median proportion within the proviral pool (0.04) (Fig. 4A) and median quantity (1.21 log copies/10^6^ PBMCs) (Fig. S1B) were not significantly different from those of other groups, although substantial heterogeneity of these values was observed between patients in each group (Fig. 4). For two PTCs, four HICs, and three CHIs, we could not detect intact provirus in the samples analyzed (Table S2), while this was not the case for PHIs. Large deletions were the most frequent defect observed in PTCs, similar to other groups of PWH, followed by both hypermutations and frameshifts (Fig. 3B). We observed a trend toward a higher proportion and number of genomes with a defect in only the *nef* gene in the HIC group compared to the proportion and number in the PTC group (*P = *0.066) (Fig. 4C and Fig. S1D), due to the presence of a similar *nef* partial deletion in every proviral sequence of two HIC participants (CODa5 and CODb1) (Table 1). Some of these proviruses had other defects, such as large deletions. This suggests that viral factors—i.e., a less fit virus—could participate in the control of infection in these two HICs. This phenomenon was not observed in PTCs. Some PWH from the PHI, CHI, and HIC groups displayed small proportions of proviruses with *nef* defects (4 to 7%), but none had a similar profile of an attenuated HIV strain. The proportion of defective proviruses among the proviral pool and their quantity per million PBMCs were significantly larger in the CHI group than in the PHI group (Fig. 4B and Fig. S1C). This appeared to be mostly explained by the large quantities of HIV DNA sequences harboring large deletions and of those harboring hypermutations in CHIs (Fig. 4D and E and Fig. S1). On the other hand, CHIs had a trend toward a lower proportion of sequences with frameshifts than PHIs (Fig. 4F); this suggests that the different genetic defects could appear at different stages of the infection and then be modulated by the dynamics of cells harboring HIV during infection. The quantities of defective proviruses were also significantly larger in CHIs than in HICs and PTCs (Fig. S1C). Globally, the quantity of defective HIV DNA was highly correlated with the total HIV DNA load (*r* = 0.968; *P* < 0.0001).

Notably, we observed identical proviral sequences (assumed to be clones [24]) for both intact and defective genomes in several participants within each group (Fig. S2), suggesting that the clonal expansion of HIV-infected cells was not dependent on the “intact” or “defective” characteristic of their provirus and could be linked to homeostatic proliferation or antigen-driven proliferation (25).

Overall, our data indicate that the control of infection in PTCs does not seem to be related to a smaller proportion or quantity of intact proviruses in these individuals than in other PWH.

### Long-term outcome of the PTC proviral landscape.

**FIG 5:**
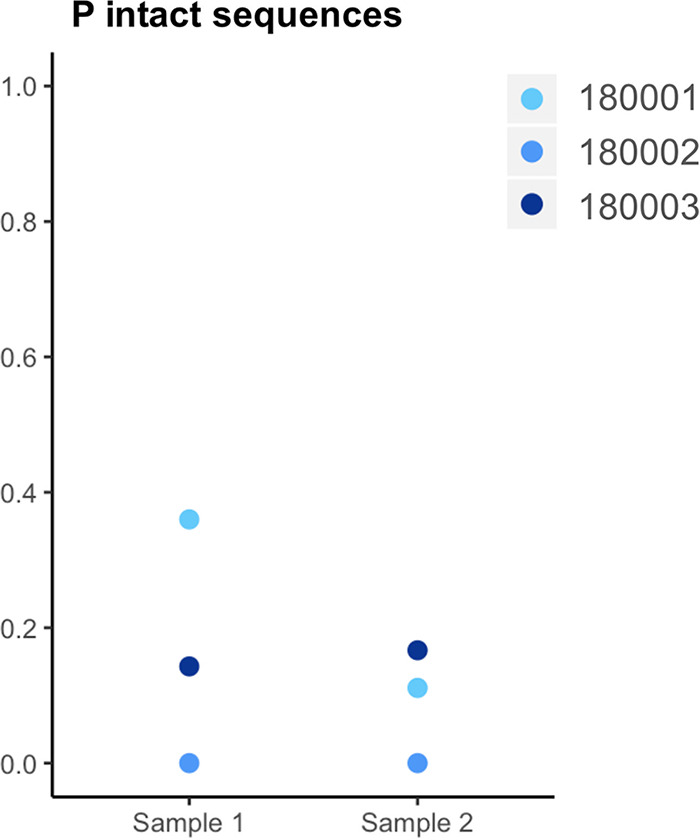
Changes in the proportions of intact full-length HIV DNA genomes for individuals with a second sample available 4 to 6 years after the initial sample. The median proportion of intact proviruses was compared between the two time points using a Wilcoxon test.

**FIG 6:**
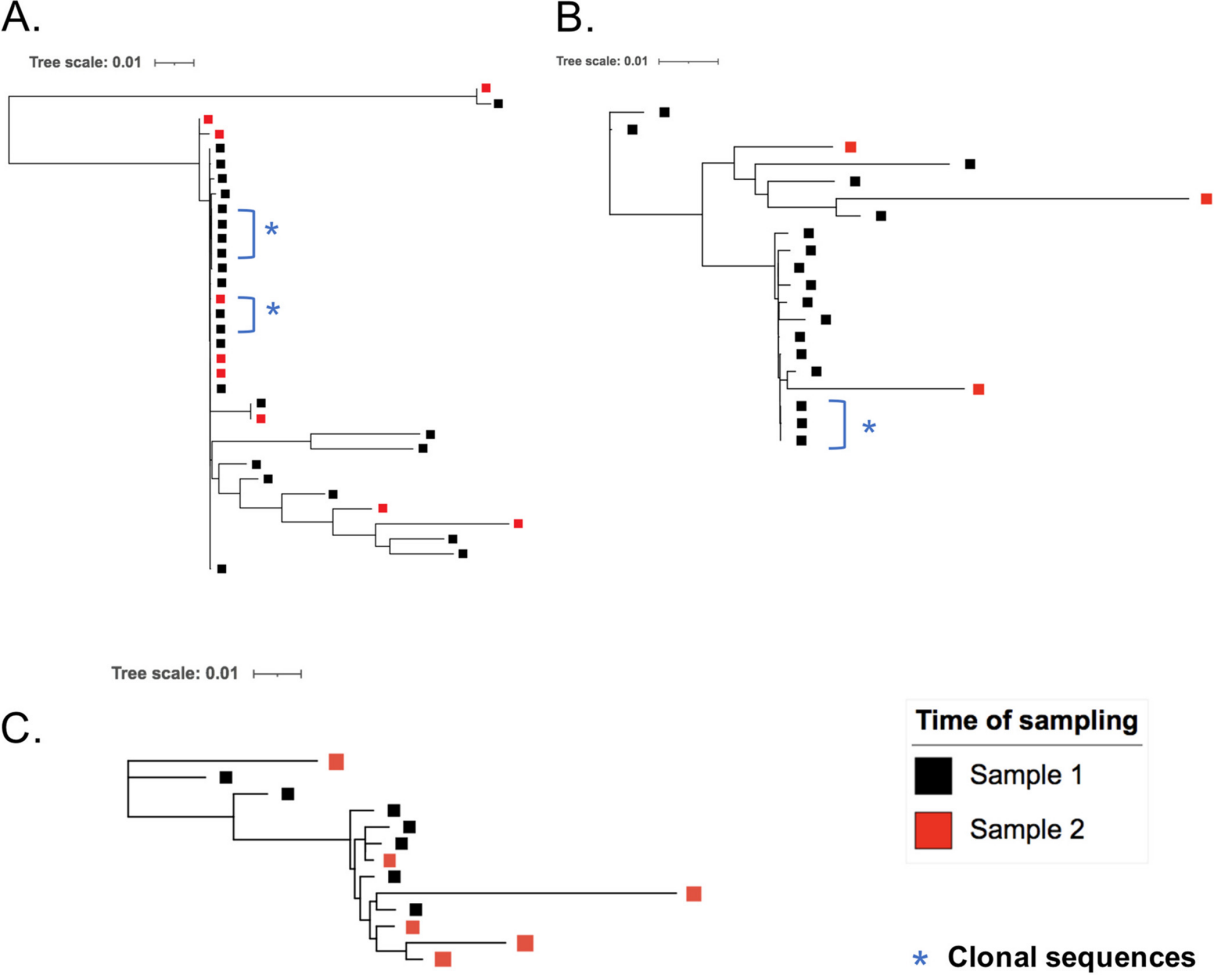
Phylogenetic trees of all nearly full-length HIV DNA genomic sequences for 3 PTCs. Individual trees of proviruses from the 3 PTCs who had two blood samples taken after TI. HIV DNA genomes were amplified from the 5′LTR to the 3′LTR and sequenced using a MiSeq instrument. Complete genomic sequences were assembled using Spades and aligned with MAFFT, and the phylogenetic tree was inferred with PhyML. Black squares represent sequences from the first samples, and red squares those from the second samples. Blue stars indicate identical sequences assumed to be clones. 180001 (A); 180002 (B); 180003 (C).

As we had access to a second sample taken 4 to 6 years after the initial sample for three individuals from the PTC group, we next investigated whether their proviral landscape had evolved or not. Two of these participants (180001 and 180002) maintained total control of viremia during the whole follow-up (ultrasensitive plasma HIV RNA loads of <8 and <10 copies/mL, respectively), while the third (180003) had begun to relapse (plasma HIV RNA load of 1,278 copies/mL) and resumed ART immediately after this sample was obtained (Table 1). One of the three individuals (180001) appeared to have a decrease in the proportion of intact proviruses, while the other two had no or little evolution of this proportion (Fig. 5 and Table S2). Details concerning the genetic defects are presented in Table S2. Phylogenetic trees of the full-length HIV DNA sequences of these 3 PTCs showed that proviral sequences from the two time points were mixed (Fig. 6), indicating that there had been no or very little evolution of the viral strains and no selective pressure. This reflects the effective control of viral replication during a long period of remission after TI.

## DISCUSSION

In the hope of inducing remission of HIV infection without the necessity for lifelong intake of antiretroviral therapy, a better understanding of the factors associated with the control of infection in PTCs, who represent models of remission, is needed. A characteristic of PTCs is a small reservoir size, which appears to be a necessary but insufficient criterion. Indeed, only a minority of individuals with a small reservoir size keep the control of infection in the case of TI (3). We describe here for the first time the viral diversity and the proportions of intact and defective proviruses in PTCs almost a decade after TI, with ultradeep sequencing technologies providing an in-depth characterization of archived genomes constituting the HIV reservoir.

One hypothesis was that low viral diversity could contribute to, and be the result of, the control of HIV infection. In our study, the PTC, PHI, and HIC-a groups presented similarly low genetic diversities, whereas those of the HIC-b and CHI groups were higher, consistent with the past viral replication. Concordantly, De Azevedo et al. showed that proviral diversity was higher in viremic controllers (with persistent viremia at 80 to 2,000 copies/mL) than in elite controllers (persistent undetectable viral loads) (16). The link between viral diversity and history of viremia is also reinforced by the significant correlation in our study between the diversity indexes and the total HIV DNA load: previous studies showed that when measured in situations of controlled infection, this biomarker of persistence reflects the history of HIV infection and cumulative viremia (5, 26). Based on this correlation, our data indicate that no or very little viral replication occurred in the HIC-a group or in the PTCs for all the years since TI. This low viral diversity reflects the sustained control of viral replication since early infection. Of note, our study also reveals substantial variability in the genetic proviral diversity among PTCs, as previously observed for other groups of PWH.

The similar results between PTCs and PHIs call into question the predictive value of viral genetic diversity for virologic control or breakthroughs. Previous studies are discordant concerning this potential predictive value. Pernas et al. showed no significant difference in *gag* gene diversity but a higher diversity of the *env* gene in cases of loss of natural control than in persistent control (27). In contrast, De Azevedo et al. found no predictive value of *env* diversity for virologic breakthroughs (28). In our study, *RT* (*pol* gene) viral diversity was not predictive of a breakthrough in the four following years in HICs or in PTCs (data not shown). Unfortunately, we did not have enough biological material to study the viral diversity of the *env* gene, which might have been more informative. In contrast, viral blips were previously shown to be predictive for the loss of control in PTCs and HICs (29, 30) and for the time before rebound after TI in early-treated individuals (9). Plasma viral loads thus appear to be better predictors of future breakthroughs than viral diversity of HIV DNA, which takes longer to be archived.

Our study also explored the proportions of intact and defective proviruses among the proviral pool during different contexts of controlled HIV infection and provided the first complete genome analysis of HIV DNA in both PTCs and HICs. Of note, the HICs for whom we obtained viral genome sequences in this study had a higher median HIV DNA level than the 202 HICs analyzed in the national ANRS-CODEX cohort (6). A peculiarity of the HIC group was the presence of a partial *nef* deletion in every proviral sequence of two HICs, which could be associated with a decrease in viral infectivity (23, 31). Our finding of *nef* defects in all sequenced proviruses suggests that either the founder virus might have been an attenuated strain in these cases or the cells harboring nonattenuated proviruses were progressively eliminated by the immune response and these proviruses with defects in *nef* became largely predominant. Concordantly, previous reports showed that several cases of spontaneous control of HIV infection were linked to the presence of attenuated viral strains harboring a truncated or deficient protein, in particular Env, Vif, or Nef (32–34). This characteristic for only two of the seven HICs, as well as the high interindividual heterogeneity of results observed within this group, highlight the plethora of mechanisms that could contribute to HIV control. No *nef* deletion was observed in PTCs in our results, suggesting that posttreatment and natural control may rely upon distinct mechanisms, although both groups have a small blood HIV reservoir (35). The lack of Nef-deficient proviruses in the PTC group should be confirmed in more individuals.

When studying the complete HIV DNA genome of PTCs, we showed that they had low proportions of intact genomes in samples taken at a median of 9.4 years after TI, not different from the proportions observed in other groups. These data suggest that other factors play a role in the viral control. High heterogeneity of the proportions of intact proviruses was observed among PTCs (0% to 36%), as observed in other groups of PWH. In their study, Sharaf et al. used single-genome amplification and next-generation sequencing (NGS) to analyze samples from before TI from 10 PTCs and 16 NCs who experienced viral rebound after TI (17). The proportions of intact genomes before TI were similar in both groups (median [range], 1.4% [0 to 42%] and 4.1 [0 to 32%] in the future PTCs and NCs, respectively, out of a median [range] of 27 [3 to 73] proviral sequences per individual for PTCs and 50 [12 to 120] for NCs). Our results show that this status is maintained for several years after TI. Sharaf’s study also revealed that PTCs have a genetically intact proviral reservoir 7-fold lower than that of NCs in absolute values before TI. This smaller number of intact viral genomes resulted from lower total HIV DNA loads in PTCs than in NCs. In our study, several years after TI, the quantity of intact proviruses was not different than that observed in aviremic PWH on ART or in HICs. Only the number of defective proviruses was lower in PTCs than in CHIs, similar to PHIs. As PTCs had been treated since the primary infection and well controlled afterwards, there was no opportunity for viral evolution and accumulation of proviruses with defects. Defective proviruses can contribute to HIV pathogenesis (5) by the production of viral RNAs and proteins/antigens that continuously stimulate the immune system (36, 37) or to infectious viruses after complementation, even if this process is probably less efficient than production by intact proviral genomes (38). This could partly explain the facts that the total HIV DNA load, which is correlated to a high level of defective proviruses, is a clinically relevant marker (5) and that a reduced number of defective proviruses could be beneficial.

Based on the presence of some proviral sequences with strict identity, assumed to be clones with identical integration sites (24), we showed that clonal cell expansion contributed to the persistence of both intact and defective proviruses, as previously described (11, 39).

Interestingly, the comparison of samples taken 4 to 6 years later (i.e., 11.8 to 15.2 years after TI) from the three PTCs in our study showed no or little evolution of the proviral landscape, either in the proportions of intact and defective genomes or in the viral sequences themselves, as shown by the intermingled sequences on the phylogenetic tree. These results suggested that the stability of the proviral landscape in PTCs between the period before TI and 1.4 years after TI that was previously described (17) is prolonged for more than 10 years after TI. These data suggest that the blood proviral landscape in PTCs is preserved early by ART and does not seem to evolve after TI due to effective control of HIV. Nevertheless, as all PHIs do not become PTCs in the case of TI (4), despite their similar proviral characteristics, host factors most probably predominate in the control of infection in cases of HIV remission. One recently suggested hypothesis is the control of viral replication by particularly efficient natural killer (NK) cells in association with HLA-Bw4 and HLA-C2 killer immunoglobulin-like receptor (KIR) ligands (40). In ART-treated PWH, Einkauf et al. recently showed that proviruses integrated in nongenic chromosomal regions were transcribed less that those in genic regions and that transcriptionally active proviruses underwent a negative selection over time, except in the case of clonal expansion (41). Such a study of integration sites of intact and defective proviruses in PTCs could provide further information on their reservoirs. We can hypothesize that the NK immune response in PTCs could have contributed to the selection of cells harboring either intact proviruses integrated in nongenic regions or defective proviruses. Control in PTCs could then be associated with a “block and lock” mechanism, which could be effective in the presence of small quantities of intact proviruses. The noticeable interindividual heterogeneity shown for the first time among PTCs for all the biomarkers we studied points out that several mechanisms of control could exist and that a single strategy for cure might not fit all PWH.

One limitation of this study is the relatively small number of sequences analyzed because of the low HIV DNA loads. The complexity of TI and the rarity of posttreatment control limit large studies on PTCs. Very few cohorts include PTCs with such a long period of time after TI, making the data from the ANRS-VISCONTI cohort very valuable. Recent data have indicated that the proportions of defective proviruses could differ between blood and tissues and that viruses that rebounded after TI could have come from diverse anatomical compartments (42). Further studies on lymphoid tissues and on animal models could be informative of the global proviral landscape in remission, including integration sites, and help in approaching the complexity of viral control.

To conclude, the present study provides for the first time an in-depth characterization of the proviral landscape in PTCs nearly a decade after TI and compares it to that of HICs. Our results show that the PTC group presents similarly small proportions and quantities of intact proviruses as the other groups examined in the study. Although host/immune factors most probably play a role in posttreatment control, the particularly small blood HIV reservoirs and the low frequencies of intact proviruses in PTCs could facilitate the control of infection in these patients. Furthermore, the low viral diversity and the absence of evolution in the landscape of the HIV DNA quasispecies after TI confirm the slow dynamics of HIV infection in PTCs. This and other studies in PTCs should help in designing strategies to achieve sustained viral remission in the absence of ART.

## MATERIALS AND METHODS

### Patients and samples.

PTCs from the ANRS-VISCONTI cohort (2) who had available frozen blood cells (5 to 8 million PBMCs) were included (*n* = 8). A second time point was analyzed when a subsequent sample was available (*n* = 3). The criteria for inclusion in the cohort were HIV-1 infection, pretherapeutic plasma viral load above 2,000 copies/mL, ART maintained for at least 12 months, and viral load below 400 copies/mL in two consecutive samples more than 1 year after TI. Natural HIV controllers were selected from the French multicenter ANRS-CODEX cohort (19) based on the availability of samples (*n* = 13). The criteria for inclusion in the cohort were as follows: HIV-1 infection with a follow-up time longer than 5 years, the last five plasma HIV RNA viral loads lower than 400 copies/mL, and no ART received. The PHI and CHI groups were selected from outpatients’ follow-up at Orleans Hospital. The PHI (*n* = 8) group was constituted of patients receiving ART continuously for more than 3 years that was initiated in primary infection and considered effective on the basis of controlled viral load (<50 copies/mL) for more than 2 years. The CHI group (*n* = 6) was constituted of patients receiving ART initiated in chronic infection and with plasma viral load peaks similar to those observed for the PHI group. None had reached the AIDS stage. All participants gave informed consent. The VISCONTI study and the CODEX cohort were approved by the ethics review committees of Ile de France VII. The study concerning other patients was approved by the Tours ethics review committee. All analyses were carried out retrospectively on cryopreserved blood cells.

### Virological quantifications.

Routine HIV RNA viral loads were quantified with the commercial assay available in each clinical center. Ultrasensitive HIV RNA quantifications were performed on 2 to 10 mL of plasma using either a generic HIV real-time PCR assay (Biocentric) or an adaptation of the COBAS Ampliprep/COBAS TaqMan HIV-1 test version 2 (Roche Diagnostics) (6). Total HIV DNA loads were quantified prospectively in the cohorts using the GENERIC HIV DNA real-time PCR assay (Biocentric), as previously described (6, 43).

### PCR and NGS analyses. (i) *RT* gene.

Viral genetic diversity, together with the presence of stop codons/hypermutations, was analyzed based on an amplicon from the *RT* region of the *pol* gene (20). Amplification was carried out as previously described by the ANRS French resistance group (HXB2 coordinates of the amplicon, 2609 to 3292) (44), with an adapted nested PCR including the multiplex identifier (MID) of the 454 GS Junior instrument (Roche Diagnostics). Deep sequencing of the *RT* amplicon was performed using GS Junior sequencing XL+ kits, which provided reads up to 800 bp long, following the manufacturer’s instructions. The 8E5/LAV cell line was included as a control.

### (ii) Near-full-length-genome sequencing.

Supplemental data on the proportion of each type of genetic defect and the global proportions of intact and defective proviruses were obtained based on a complete genome analysis. For single-genome analyses (SGAs), limiting dilution conditions, determined separately for each sample, were used for the first PCR in order to avoid the preferential amplification of short fragments and to allow the study of viral haplotypes. HIV DNA was then amplified from the 5′LTR to the 3′LTR with nested PCR using PrimeSTAR GXL DNA polymerase (TaKaRa) and previously described primers (36). The following PCR conditions were applied: 98°C for 10 min, followed by 98°C for 10 s, 58°C or 60°C (for the first and second PCR, respectively) for 15 s, and 68°C for 9 min for 40 cycles, and then 10°C indefinitely. The PCR mixture volume was 50 μL. Five microliters of the first PCR mixture was used in the second-round PCR. Positive nested PCR products were identified by electrophoresis on a 1% agarose gel and purified with either a QIAquick PCR Purification Kit or a QIAquick Gel Extraction Kit (Qiagen). Library preparations were performed using a Nextera DNA Flex Library Prep Kit, and paired-end sequencing (2 × 250 bp) was performed using a MiSeq Reagent Nano Kit on a MiSeq instrument (Illumina) following the manufacturer’s instructions. As a quality control, we performed the same PCRs with limiting dilution on an 8E5/LAV cell line, which was sequenced three times in the first Illumina run for repeatability verification and then once in each run for reproducibility assessment.

### Bioinformatics analyses.

**(i) 454 data.** Reads were analyzed with the Roche software AVA. After passing quality filters, haplotypes were aligned with Clustal using HXB2 as a reference and submitted to the online Los Alamos HIV Database Hypermut tool for the identification of hypermutated sequences (45).

To estimate the viral diversity, the mean branch length was calculated for each participant using Gotree (46) after construction of a phylogenetic tree using PhyML 3.3.2018062 (47) (options -m GTR -f m -c 4 -a e -o tlr), including nondefective sequences only. To avoid creating bias in the results because of the variability in the number of reads obtained for each sample, we used a rarefaction approach to calculate the different diversity indexes (16). A random sampling of 20 sequences without replacement was performed 1,000 times; the final values are the means of these 1,000 repetitions. With this approach, we calculated the mean number of alleles (or variants) per nucleotide site, the mean entropy, and the *p*-distance.

**(ii) Illumina data.** Quality control of FASTQ files was performed using FastQC version 0.11.8 and MultiQC version 1.7 (48). After masking parts of the HXB2 reference genome corresponding to the primers, reads were mapped to HXB2 using Minimap2 version 2.14 (49) with the option “-ax splice.” SAM files were converted to BAM files and indexed using SAMtools version 1.9 (50). In parallel, sequences were assembled using Spades version 3.11.1 (51) at different coverage cutoffs (going from 0 to 200) with the options “--cov-cutoff <cutoff> --careful”. Premature stop codons due to hypermutations and frameshifts were detected in HIV genes extracted from these assembled sequences using pairwise alignment implemented in Goalign version 0.3.0-alpha (46).

To build the phylogenetic tree, sequences were first aligned with MAFFT version 7.313 (52). Then, sequences having more than 80% gaps and alignment columns having at least one gap were removed using Goalign version 0.3.1 (command goalign clean). The final alignment contained 192 sequences of 6,376 nucleotides. This alignment was reformatted into Phylip format (command goalign reformat phylip), and duplicate sequences were temporarily removed (command goalign dedup). The phylogenetic tree was then inferred with PhyML version 3.3.20180621 (options -m GTR -f m -c 4 -a e -o tlr), and duplicate sequences were reintroduced into the inferred tree using Goalign version 0.3.1 (command Goalign repopulate). The final tree was then uploaded to iTOL along with its annotations produced with table2itol (https://github.com/mgoeker/table2itol).

### Quality controls for the NGS analyses.

To verify the reproducibility of the NGS and bioinformatic analyses, the 8E5/LAV cell line was included as a control in both *RT* and full-genome analyses. All *RT* sequences belonged to a unique haplotype, harboring no hypermutations or stop codon mutations (data not shown). For the full-genome analysis, the exact same sequence was found for the assembled 8E5/LAV contig of every run and of the three replicates of the first run, validating the NGS process. Phylogenetic trees of all *RT* sequences and all complete genome sequences confirmed the absence of cross-contamination between samples (data not shown).

### Statistics.

Statistical analyses were performed using RStudio software (version 1.0.136). Variables were compared between groups using Wilcoxon tests. *P* values of <0.05 were considered significant. Correlations were determined using Spearman’s correlation coefficients. Samples with an insufficient number of sequences obtained (<20 sequences or <4 haplotypes for the *RT* amplicon; <7 sequences for the full-length genome) were excluded (Tables S1 and S2). HIV DNA and HIV RNA viral loads above or below the limits of quantification were set to the threshold values for statistical analyses.

### Data availability.

*RT* haplotype sequences are available in GenBank under accession numbers OP994861 to OP995430. Full-length HIV DNA sequences are available in GenBank under accession numbers OP994351 to OP994860.
