# Supplementary material for: In-Depth Characterization of Full-Length Archived Viral Genomes after Nine Years of Posttreatment HIV Control
Source: Microbiol Spectr. 2023 Jan 24;11(1):e03267-22. doi: 10.1128/spectrum.03267-22 (PMC9927157; doi:10.1128/spectrum.03267-22)
Supplement: Supplemental file 1 — Supplemental material. Download spectrum.03267-22-s0001.pdf, PDF file, 1.6 MB [file spectrum.03267-22-s0001.pdf]

Supplemental material:

S1 Table. Detailed results of NGS data of the Reverse Transcriptase gene

Group		Sample	Viral subtype	n sequences (sequencing depth)	Total n haplotypes	Stop codons / hypermutations	Mean tree length *	Mean n alleles per nucleotide site **	Mean entropy **	Mean p-distance **
PTC		180001	CRF02	3192	70	yes	0.00087842	1.00777	0.007646	0.00326218
PTC		180001b	CRF02	NA	NA	NA	NA	NA	NA	NA
PTC		180002	RF with subtype A	6123	41	no	0.00073361	1.00824	0.006095	0.00348987
PTC		180002b	RF with subtype A	NA	NA	NA	NA	NA	NA	NA
PTC		180003	B	1993	72	no	0.00074998	1.01781	0.008284	0.00605549
PTC		180003b	B	NA	NA	NA	NA	NA	NA	NA
PTC		098001	B	1949	21	no	0.00131117	1.01647	0.007862	0.00615052
PTC		070001	B	22	3	no	NA	1.00296	0.001997	0.00197676
PTC		041001	B	711	4	no	0.00087756	1.0007	0.000479	0.000650608
PTC		200001	B	1925	8	no	0.00080544	1.00442	0.002582	0.00254024
PTC		073001	H	136	23	yes	0.00209805	1.03357	0.012776	0.010438
HIC	HIC-a	CODa1	B	24	7	no	0.00114898	1.01182	0.00696	0.00522884
HIC	HIC-a	CODa2	BF	357	8	yes	NA	1.00027	0.00018	0.000267062
HIC	HIC-a	CODa3	B	57	4	no	0.00103917	1.00345	0.002326	0.00243432
HIC	HIC-a	CODa4	B	185	12	no	0.00072258	1.00762	0.004378	0.00355525
HIC	HIC-a	CODa5	CRF13	NA	NA	NA	NA	NA	NA	NA
HIC	HIC-a	CODa6	CRF13	NA	NA	NA	NA	NA	NA	NA
HIC	HIC-b	CODb1	B	69	18	no	0.00122015	1.02092	0.008881	0.00615229
HIC	HIC-b	CODb2	CRF11	344	42	no	0.0008863	1.0229	0.010912	0.00787702
HIC	HIC-b	CODb3	B	22	4	yes	NA	NA	NA	NA
HIC	HIC-b	CODb4	CRF02	174	14	no	0.00082611	1.00766	0.004345	0.00349531
HIC	HIC-b	CODb5	B	29	6	no	0.00166059	1.01182	0.00697	0.00591558
HIC	HIC-b	CODb6	B	97	8	no	0.00189039	1.01611	0.056057	0.00873592
HIC	HIC-b	CODb7	C	NA	NA	NA	NA	NA	NA	NA
PHI		PHI1	B	3281	10	no	0.00078708	1.00341	0.002008	0.00203002
PHI		PHI2	B	515	8	no	0.00082996	1.00679	0.018946	0.004134
PHI		PHI3	B	56	3	no	NA	1.00442	0.002982	0.00295401
PHI		PHI4	B	744	18	no	0.00074462	1.00824	0.013148	0.00439994
PHI		PHI5	B	2022	53	yes	0.00071786	1.00922	0.00491	0.0039025
PHI		PHI6	B	380	11	yes	0.00105746	1.00442	0.030965	0.0026683
PHI		PHI7	B	119	14	no	0.0013326	1.01685	0.007646	0.00562749
PHI		PHI8	B	209	8	no	0.0007144	1.00495	0.002574	0.00234525
CHI		CHI1	B	126	12	no	0.00109103	1.01425	0.05691	0.00493933
CHI		CHI2	B	18	4	no	0.00250173	NA	NA	NA
CHI		CHI3	B	21	8	no	0.00248233	1.02515	0.014	0.010672
CHI		CHI4	B	185	42	yes	0.00122977	1.03397	0.013867	0.00922563
CHI		CHI5	B	49	6	yes	0.00178683	1.00786	0.004614	0.00440603
CHI		CHI6	B	20	8	no	0.00168481	1.01778	0.01	0.00783069

When available, the second sample from the same individual was identified with the letter “b” (e.g. 180001b is the second sample from individual 180001).
* Samples with less than 4 non-defective haplotypes were excluded.
** Mean values for 20 non-defective sequences (random sampling without replacement, 1000 repetitions).
RT haplotype sequences are available on GenBank under accession numbers OP994861 to OP995430.

S2 Table. Detailed results of SGA-NGS data of the near-full-length genome

Group		Sample	Viral subtype	n sequences	n clonal sequences (n clones)	n intact sequences	n sequences with *nef* defect	n sequences with large deletion	n sequences with frameshift	n sequences with hypermutations	n sequences with 5'LTR defect
PTC		180001	CRF02	25	6 (2)	9	0	15	0	1	0
		180001b		9	0	1	0	7	0	1	0
		180002	RF with subtype A	17	3 (1)	0	0	6	3	5	3
		180002b		4	0	0	0	1	0	3	0
		180003	B	7	0	1	0	3	2	1	0
		180003b		6	0	1	0	3	0	2	0
		098001	B	22	7 (3)	0	0	21	0	1	0
		070001	B	23	6 (1)	1	0	20	1	0	1
		041001	B	5 *	NA	NA	NA	NA	NA	NA	NA
		200001	B	4 *	NA	NA	NA	NA	NA	NA	NA
		073001	H	NA	NA	NA	NA	NA	NA	NA	NA
HIC	HIC-a	CODa1	B	NA	NA	NA	NA	NA	NA	NA	NA
		CODa2	BF	NA	NA	NA	NA	NA	NA	NA	NA
		CODa3	B	NA	NA	NA	NA	NA	NA	NA	NA
		CODa4	B	NA	NA	NA	NA	NA	NA	NA	NA
		CODa5	CRF13	9	0	0	3	5	1	0	0
		CODa6	CRF13	19	2 (1)	0	0	19	0	0	0
	HIC-b	CODb1	B	21	0	0	8	10	3	0	0
		CODb2	CRF11	NA	NA	NA	NA	NA	NA	NA	NA
		CODb3	B	18	0	7	1	10	0	0	0
		CODb4	CRF02	14	0	7	1	5	0	1	0
		CODb5	B	NA	NA	NA	NA	NA	NA	NA	NA
		CODb6	B	12	5 (2)	2	0	10	0	0	0
		CODb7	C	8	4 (1)	0	0	8	0	0	0
PHI		PHI1	B	3 *	NA	NA	NA	NA	NA	NA	NA
		PHI2	B	22	12 (2)	1	0	10	10	1	0
		PHI3	B	20	0	7	1	8	2	2	0
		PHI4	B	4 *	NA	NA	NA	NA	NA	NA	NA
		PHI5	B	19	5 (2)	8	1	9	0	1	0
		PHI6	B	25	0	8	1	14	1	1	0
		PHI7	B	31	2 (1)	4	0	23	2	1	1
		PHI8	B	35	11 (2)	5	0	18	10	1	1
CHI		CHI1	B	23	8 (3)	0	0	21	0	2	0
		CHI2	B	24	5 (2)	1	1	21	0	1	0
		CHI3	B	19	0	0	0	14	1	4	0
		CHI4	B	21	0	2	0	14	1	4	0
		CHI5	B	29	2 (1)	4	0	24	0	1	0
		CHI6	B	28	0	0	0	23	0	4	1

When available, the second sample from the same individual was identified with the letter “b” (e.g. 180001b is the second sample from individual 180001).

* Samples with less than 7 complete genome sequences were excluded from further analyses.

Full-length HIV DNA sequences are available on GenBank under accession numbers OP994351 to OP994860.

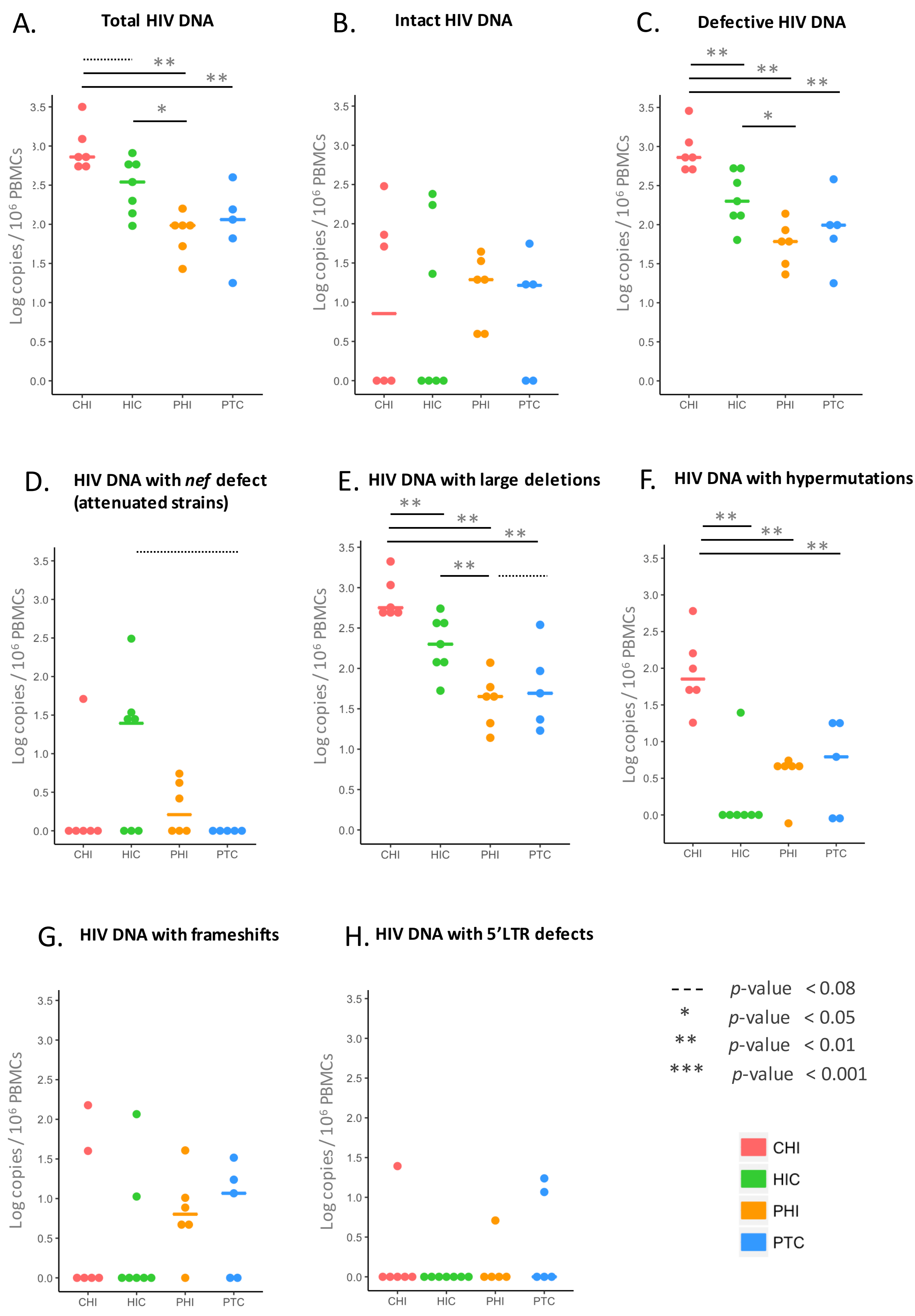

A. Total HIV DNA
B. Intact HIV DNA
C. Defective HIV DNA
D. HIV DNA with *nef* defect (attenuated strains)
E. HIV DNA with large deletions
F. HIV DNA with hypermutations
G. HIV DNA with frameshifts
H. HIV DNA with 5'LTR defects
Log copies / 10⁶ PBMCs
CHI
HIC
PHI
PTC
--- p-value < 0.08
* p-value < 0.05
** p-value < 0.01
*** p-value < 0.001

S1 Fig. HIV DNA loads for each type of provirus

A. Total HIV DNA loads (log copies/10^6 PBMCs) in each of the four groups of HIV-infected individuals.
B-H. Near-full-length HIV DNA genomes were amplified and sequenced to identify the genetic defects. The proportion of each type of provirus was multiplied by the total HIV DNA load and then log-transformed to determine the amounts of intact HIV DNA (B), defective HIV DNA (C), proviruses with only *nef* defects (attenuated strains) (C), proviruses with large deletions (E), proviruses with APOBEC-induced hypermutations (F), proviruses with frameshifts (G), and proviruses with defect on the Ψ/MSD regions of the 5'LTR (H). The mean value for each group is depicted with a colored horizontal line. The viral loads of each type of provirus were compared among groups using Wilcoxon tests. Significant differences (*p*-values <0.05) are depicted with continuous lines above the graphs, while trends (*p*-values between 0.05-0.08) are depicted with dotted lines.

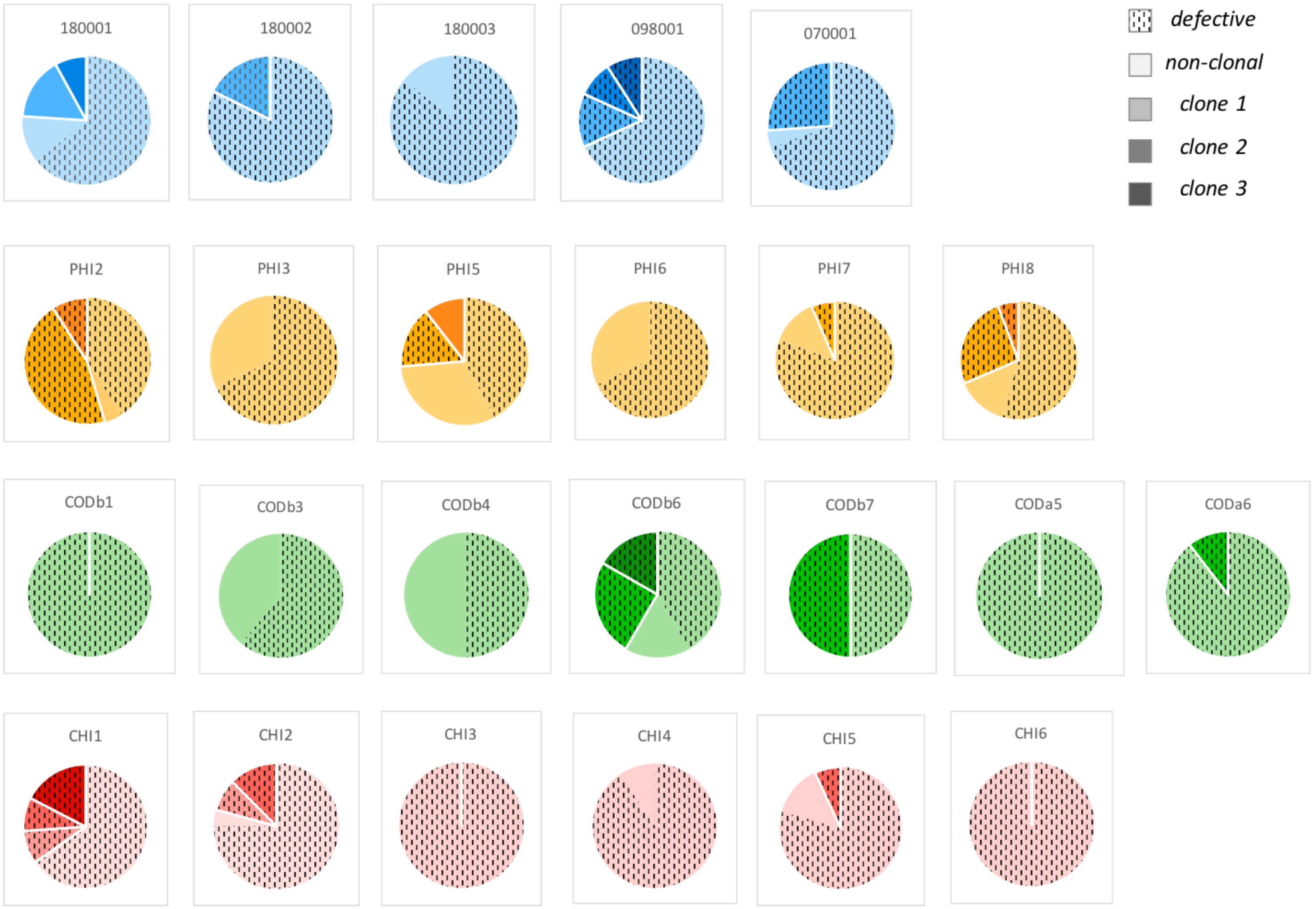

S2 Fig. Clonality of provirus sequences

Identical near-full-length genomes were assumed to be clones. No mismatch was allowed. For each group of individuals, the lighter color represents the nonclonal sequences, while darker shades represent clones. Dashed areas represent defective sequences. Notably, CODb1 and CODa5 were the two individuals harboring partially *nef*-deleted virus attenuated strains.

S1 Text. Lists of the ANRS-VISCONTI cohort and ANRS-CODEX cohort study groups

- <u>ANRS-VISCONTI study group members</u>

Faroudy Boufassa, Sylvie Orr, *CMG, Hôpital Bicêtre, Le Kremlin Bicêtre.*
Firouzé Bani-Sadr, Maxime Hentzien, Jean-Luc Berger, Isabelle Kmiec, *Maladies Infectieuses, CHU - Hôpital Robert Debré, Reims*.
Gilles Pichancourt, Safa Nasri, *Hémato-Oncologie, Centre Hospitalier Henri Duffaut, Avignon*.
Gilles Hittinger, Véronique Lambry, Anne-Cécile Beaudrey, *Maladies Infectieuses, CHITS Hôpital Sainte Musse, Toulon.*
Gilles Pialoux, Julie Chas, Christia Palacios, Anne Adda*, Maladies Infectieuses, Hôpital Tenon, Paris.*
Jean Paul Viard, Marie-Josée Dulucq, *Centre de Diagnostic et Thérapeutique, Hôtel Dieu, Paris.*
Laurence Weiss, Marina Karmochkine, Mohamed Meghadecha, *Service d'Immunologie Clinique, Hôpitaux Universitaires Paris Centre – Hôtel Dieu.*
Dominique Salmon-Ceron, Marie-Pierre Piétri, *Unité fonctionnelle de Pathologie Infectieuse, Hôtel Dieu, Paris.*
Philippe Blanche, *Médecine Interne, Hôpital Cochin, Paris.*
Jean-Michel Molina, Olivier Taulera, Diane Ponscarme, Jeannine Delgado Bertaut, *Maladies Infectieuses, Saint-Louis, Paris.*
Djamila Makhloufi, Matthieu Godinot, Valérie Artizzu, *Immunologie Clinique, Hôpital Edouard Herriot, Lyon.*
Patrick Miailhes, Laurent Cotte, Sophie Pailhes, Anne Conrad, Ludovic Karkowski, Stanislas Ogoudjobi, *Maladies Infectieuses et Tropicales, Hôpital de la Croix Rousse, Lyon.*
Yazdan Yazdanpanah, Sophie Matheron, Cindy Godard, *Maladies Infectieuses, Bichat, Paris.*
Louis Bernard, Frédéric Bastides, Olivier Bourgault, *Maladies Infectieuses, Hôpital Bretonneau, Tours.*
Christine Jacomet, Emilie Goncalves, *Maladies Infectieuses, Hôpital Gabriel-Montpied, Clermont-Ferrand.*
Pascal Chavanet, Lionel Piroth, Sandrine Gohier, *Infectiologie, CHU François Mitterrand, Dijon.*
Agnès Meybeck, Thomas Huleux, Pauline Cornavin, *Maladies Infectieuses, Hôpital Gustave Dron, Tourcoing.*
Yasmine Debab, David Théron, *Maladies Infectieuses et Tropicales, Hôpital Charles Nicolle, Rouen.*
Thierry Prazuck, Laurent Hocqueloux, Barbara De Dieuleveult, *Maladies Infectieuses, Hôpital de La Source, Orléans.*

- <u>ANRS-CODEX study group members</u>

Dr Jean-Pierre Faller, Mme Patricia Eglinger, *Service des Maladies Infectieuses, CH de Belfort-Montbéliard, Belfort*.
Pr Pascal Roblot, M. David Plainchamp, *Service de Médecine Interne, CHU Poitiers-La Milétrie, Poitiers*.
Dr Hugues Aumaître, Mme Martine Malet*, Service des Maladies Infectieuse et Tropicales, CH de Perpignan, Perpignan*.
Dr Christine Rouger, Pr Gérard Rémy, Melle Kmiec Isabelle, *Service des Maladies Infectieuses, CHU Reims-Hôpital Robert Debré, Reims.*
Dr Jean-Luc Delassus, *Service de Médecine Interne, CHI Ballanger, Aulnay Sous-Bois*.
Dr Alain Devidas, *Service d'Hématologie, CH Sud-Francilien – Hôpital Gilles de Corbeil, Corbeil – Evry*.
Dr Eric Froguel, Mme Sylvie Tassi, *Service de Médecine Interne-Maladies Infectieuses, CH de Marne la Vallée, Jossigny.*
Dr Philippe Genet, Mme Juliette Gerbe, *Service Hématologie-Immunologie, Centre Hospitalier Victor Dupouy, Argenteuil*.
Pr Olivier Patey, Mr Richier Laurent, *Service des Maladies Infectieuses et Tropicales, CHI Villeneuve Saint Georges, Villeneuve Saint Georges.*
Dr Marie-Christine Drobacheff, Dr Aurélie Proust, *Service de Dermatologie, Hôpital Saint-Jacques, Besançon*.
Dr Helder Gil, *Service de Médecine interne, CHU de Besançon, Besançon.*
Dr Laurence Gérard, Pr Eric Oksenhendler, *Service d'Immunopathologie clinique* ; Pr Jean-Michel Molina, Dr Caroline Lascoux, Mme Sylvie Parlier, *Service de Maladies Infectieuses et Tropicales ; Hôpital Saint Louis, Paris.*
Pr Frédéric Lucht, Mme Véronique Ronat, *Service de Maladie Infectieuse, Hôpital Bellevue, Saint Etienne.*
Pr Michel Dupon, Dr Hervé Dutronc, Mme Séverine Le Puil, Pr Didier Neau, *Service des Maladies Infectieuses, CHU- Hôpital Pellegrin, Bordeaux.*

Pr Patrick Mercié, *Service Tropicales ;* Pr Philippe Morlat, Mme Sabrina Caldato, Pr Jean-Luc Schmit, Mme Nathalie Decaux, *Service de Médecine Interne et maladies tropicales ; CHU – Hôpital Saint André, Bordeaux.*
Dr Jean-Pierre Bru, Mme Gaëlle Clavere, *Service des Maladies Infectieuses, Centre Hospitalier Annecy, Annecy.*
Pr Olivier Lambotte, Pr Jean-François Delfraissy, Pr Cécile Goujard, Mme Katia Bourdic, *Service de Médecine Interne* ; Pr. Daniel Vittecoq, Mme Claudine Bolliot, *Service des Maladies Infectieuses* ; Dr Thierry Lambert, *Consultation d'Hématologie ; AP-HP – CHU de Bicêtre, Le Kremlin Bicêtre.*
Pr Jean-François Bergmann, Mme Maguy Parrinello, *Service de Médecine Interne A, Hôpital Lariboisière, Paris.*
Dr Gilles Pichancourt, *Service Hématologie, Hôpital Henri Duffaut, Avignon.*
Dr Yves Welker, *Service de maladies Infectieuses, CHI de Poissy-Saint Germain en Laye, Saint Germain en Lay.*
Dr Alain Lafeuillade, Mme Philip Gisèle, *Service d'Infectiologie, CHITS Hopital Sainte Musse, Toulon.*
Pr Christophe Rapp, Melle Lerondel, *Service des Maladies Infectieuses, Hôpital d'Instruction des Armées Bégin, Saint Mandé.*
Dr Pierre de Truchis, Mme Huguette Berthe, *Département de Médecine Aigue Spécialisée, Hôpital Raymond Poincaré, Garches.*
Dr Vincent Jeantils, Mme Fatouma Mchangama, *Unité de Maladies Infectieuses, Hôpital Jean Verdier, Bondy.*
Dr Paul Henri Consigny, Mme Fatima Touam, *Consultation de Maladies Infectieuses, Centre Médical de l'Institut Pasteur, Paris.*
Pr Gilles Pialoux, Mme Sophie le Nagat, *Service des Maladies Infectieuses, Hôpital Tenon, Paris.*
Pr Olivier Bouchaud, Mme Patricia Honoré, *Service de Médecine Interne et Endocrinologie, Hôpital Avicenne, Bobigny.*
Pr François Boué, Mme Mariem Raho-Moussa, *Service de Médecine Interne, Hôpital Antoine Béclère, Clamart.*
Dr Jean-Paul Viard, Mme Agnès Cros, Pr Dominique Salmon-Céron, Mme Marie-Pierre Pietri, Pr Laurence Weiss, Dr Lio Collias, *Consultation d'Immunologie Clinique et Infectiologie, Hôpital Hôtel Dieu, Paris.*
Dr David Zucman, Pr Olivier Blétry, Mme Dominique Bornarel, *Service de Médecine Interne, Hôpital Foch, Suresnes.*
Dr Emmanuel Mortier, Mme Zeng Feng, *Service de Médecine Interne, Hôpital Louis Mourier, Colombes.*
Pr Jean-Daniel Lelièvre, *Service d'Immunologie Clinique, Hôpital Henri Mondor, Créteil.*
Pr Christine Katlama, Mme Yasmine Dudoit, Dr Anne Simon, Mme Catherine Lupin, *Service des Maladies Infectieuses, Hôpital Pitié-Salpêtrière, Paris.*
Pr Pierre-Marie Girard, Mme Michèle Pauchard, *Service des Maladies Infectieuses, Hôpital Saint Antoine, Paris.*
Dr Sylvie Abel, Dr André Cabié, *Service de Maladies Infectieuses et Tropicales, Hôpital Pierre Zobda-Quitman, Fort de France, Martinique.*
Dr Pascale Fialaire, Dr Jean-Marie Chennebault, M Sami Rehaiem, *Service des Maladies Infectieuses et Tropicales, CHU Angers, Angers*.
Dr Luc de Saint Martin, Dr Perfezou, M Jean-Charles Duthe, *Service de Pneumologie, CHU de Brest, Brest.*
Pr Pierre Weinbreck, Dr Claire Genet, *Service des Maladies Infectieuses, CHU de Limoges, Limoges.*
Dr Djamila Makhloufi, Mme Florence Garnier, *Service d'Immunologie clinique* ; Dr Patrick Miailhes, M Stanislas Ogoudjobi, *Service de Maladies Infectieuses et Tropicales ; HCL – Hôpital Edouard Herriot, Lyon.*
Dr Isabelle Poizot-Martin, Dr Olivia Fauche, Mme Alena Ivanova, *Service Hématologie-CISIH, Hôpital Sainte Marguerite, Marseille*.
Dr Patrick Philibert, Mme Mame Penda Sow, *Consultation de Médecine Interne, Hôpital Européen Marseille, Marseille.*
Pr Patrick Yeni, Dr Sophie Matheron, Mme Godard Cyndi, *Service des Maladies Infectieuses, Hôpital Bichat Claude Bernard, Paris.*
Pr François Raffi, Mr Hervé Hüe, *Service de Médecine Interne, Hôpital de l'Hôtel Dieu, Nantes.*
Dr Philippe Perré, *Service de Médecine Interne post-Urgence, Centre Hospitalier Départemental, La Roche sur Yon.*
Pr Pierre Marie Roger, Mme Aline Joulie*, Service des Maladies Infectieuses* ; Pr Éric Rosenthal, *Service Médecine Interne ; CHU – Hôpital l'Archet, Nice.*
Pr Christian Michelet, Dr Faouzi Souala, Mme Maja Ratajczak, *Service des Maladies Infectieuses, CHU-Hôpital Pontchaillou, Rennes.*
Dr Marialuisa Partisani, Mme Patricia Fischer, *HUS-Hôpital Civil, Strasbourg.*
Pr Louis Bernard, Mme Pascale Nau, *Service des Maladies Infectieuses, CHRU – Hôpital Bretonneau, Tours.*
Pr Pierre Delobel, Mme Florence Balsarin, *Service des Maladies Infectieuses, CHU – Hôpital Purpan, Toulouse.*
Dr Marc De Lavaissiere, *Service Médecine Interne, CHG de Montauban, Montauban.*
Pr Renaud Verdon, Mr Philippe Feret, *Service des Maladies Infectieuses, CHU – Hôpital de la Côte de Nacre, Caen.*
Dr Christine Jacomet, *Service des maladies Infectieuse, CHU Gabriel Montpied, Clermont Ferrand.*
Dr Lionel Piroth, Mme Sandrine Gohier, *Service de Maladies Infectieuses et Tropicales, CHU – Hôpital du Bocage, Dijon.*
Dr Pascale Leclercq, Mme Gerberon, *Service Médecin Aigue spécialisée, CHU-Hôpital Albert Michallon, Grenoble.*

Dr Agnés Meybeck, Dr Raphaël Biekre, *Service des Maladies Infectieuses, CH – Hôpital Gustave Dron, Tourcoing*.
Pr Thierry May, Mme Bouillon*, Service de maladies Infectieuses et tropicales, CHU Nancy, Nancy.*
Pr François Caron, Dr Yasmine Debab, M David Theron, *Service de maladies Infectieuses et tropicales, CHU – Hôpital Charles Nicolle, Rouen.*
Dr Marc Gatfosse, *Service de Médecine Interne, CH René Arbeltier, Coulommiers.*
Dr Martin Martinot, Mme Anne Pachart, *Service de Maladies Infectieuses-Médecine Interne, Hôpitaux Civils de Colmar, Colmar.*
Dr Patrice Poubeau, *Service de Pneumo-phtisiologie, Centre Hospitalier Sud Réunion - Hôpital de St Pierre, Saint Pierre, La Réunion*.
Dr Catherine Gaud, *Service Immunologie Clinique, Centre Hospitalier Félix Guyon, Ile de la Réunion.*
Dr Agnès Uludag, *Service de Médecine Interne, Hôpital Beaujon, Clichy.*
Dr Philippe Arsac, Mme Lydia Bouaraba, *Service de Médecine Interne, CHR Orléans- Hôpital Porte Madeleine, Orléans.*
Dr Laurent Hocqueloux, Mme Barbara de Dieulevault, *Service de Maladies Infectieuses et Tropicales, Hôpital Orléans la Source, Orléans.*
Dr Isabelle De Lacroix Szmania, M Laurent Richier, *Service des Médecine Interne, Centre Hospitalier Intercommunal, Créteil.*
Dr Vincent Daneluzzi, *Service de Médecine A, CASH – Hôpital Max Fourestier, Nanterre*.
Dr Elisabeth Rouveix, *Service de Médecine Interne 2, Hôpital Ambroise Paré, Boulogne.*
Dr Geneviève Beck-Wirth, *Service d'Hématologie Clinique VIH, Centre Hospitalier de Mulhouse, Mulhouse.*
Dr Philippe Romand, *Service de Pneumologie, CHI Les Hôpitaux du Léman, Thonon les Bains.*
Dr Laurent Blum, Mme Martine Deschaud, *Service Médecine-Gastroentérologie, Centre hospitalier René Dubos, Pontoise.*
Dr Christophe Michau, *Service de Médecine Interne, Centre Hospitalier de Saint Nazaire, Saint Nazaire.*
Dr Christian Bernard, Mme Florence Salaun, *Service de Médecine Interne, CHR Metz Thionville – Hôpital Notre Dame de Bon Secours, Metz*.
Dr Philippe Muller, *Service de Dermatologie, Hôpital Beauregard, Thionville.*
Dr Yves Poinsignon, *Service de Médecine Interne, Hôpital Prosper Chubert, CHBA, Vannes.*
Dr Annie Lepretre, Mme Martine Deschaud, *Service de Médecine Interne, Hôpital Simone Veil, Eaubonne.*
Pr Albert Sotto, Mme Doncesco, *Service des Maladies Infectieuses et Tropicales, CHU Caremeau, Nîmes.*
Dr Pascale Perfezou, M Jean Charles Duthe, *Service de Pneumologie, CH de Cornouaille – Hôpital Laennec, Quimper*.
Dr Mathilde Aurore Niault, Mme Virginie Mouton- Rioux, *Service d'hématologie, maladie Infectieuses, CH Bretagne Sud, Lorient.*
Dr Jean-Philippe Talarmin, M Jean Charles Duthé, *Service Médecine Interne, CH de Cornouaille – Hôpital Laennec, Quimper.*
Dr Dupont Mathilde, M Stéphane Natur, *Service des Maladies Infectieuses et Tropicales, CH Saint Malo, Saint Malo.*
Dr Hikombo Hitoto, M Ali Mahamadou Ibrahim, *Service de Maladies Infectieuses et tropicales, Centre Hospitalier Le Mans, Le Mans.*
